# Evolutionary plasticity determination by orthologous groups distribution

**DOI:** 10.1186/1745-6150-6-22

**Published:** 2011-05-17

**Authors:** Rodrigo JS Dalmolin, Mauro AA Castro, José L Rybarczyk Filho, Luis HT Souza, Rita MC de Almeida, José CF Moreira

**Affiliations:** 1 Department of Biochemistry, Institute of Basic Health Sciences, Federal University of Rio Grande do Sul, Rio Grande do Sul, Brazil; 2 Department of Physics, Institute of Physics, Federal University of Rio Grande do Sul, Rio Grande do Sul, Brazil

## Abstract

**Background:**

Genetic plasticity may be understood as the ability of a functional gene network to tolerate alterations in its components or structure. Usually, the studies involving gene modifications in the course of the evolution are concerned to nucleotide sequence alterations in closely related species. However, the analysis of large scale data about the distribution of gene families in non-exclusively closely related species can provide insights on how plastic or how conserved a given gene family is. Here, we analyze the abundance and diversity of all Eukaryotic Clusters of Orthologous Groups (KOG) present in STRING database, resulting in a total of 4,850 KOGs. This dataset comprises 481,421 proteins distributed among 55 eukaryotes.

**Results:**

We propose an index to evaluate the evolutionary plasticity and conservation of an orthologous group based on its abundance and diversity across eukaryotes. To further KOG plasticity analysis, we estimate the evolutionary distance average among all proteins which take part in the same orthologous group. As a result, we found a strong correlation between the evolutionary distance average and the proposed evolutionary plasticity index. Additionally, we found low evolutionary plasticity in *Saccharomyces cerevisiae *genes associated with inviability and *Mus musculus *genes associated with early lethality. At last, we plot the evolutionary plasticity value in different gene networks from yeast and humans. As a result, it was possible to discriminate among higher and lower plastic areas of the gene networks analyzed.

**Conclusions:**

The distribution of gene families brings valuable information on evolutionary plasticity which might be related with genetic plasticity. Accordingly, it is possible to discriminate among conserved and plastic orthologous groups by evaluating their abundance and diversity across eukaryotes.

**Reviewers:**

This article was reviewed by Prof Manyuan Long, Hiroyuki Toh, and Sebastien Halary.

## Background

Biological systems are constantly changing at different hierarchical levels, such as genome sequences, gene/protein networks and organismal phenotypes. However, evolutionary constraints selectively act on all levels of organization allowing some changes and constraining others. Regarding specifically genomes, constraints do not act equally among all genetic sequences. Different classes of organisms (*e.g. *prokaryotes, unicellular eukaryotes, and multicellular eukaryotes) as well as different genomes structures (*e.g. *codifying sequences, introns, and "junk" sequences) can present huge differences in constraints. Even among codifying sequences, constraints act differently depending on the effect a possible mutation will generate on gene product. Synonymous mutations, for instance, are less constrained comparing to non-synonymous mutations. In addition, mutations in gene regions responsible for crucial sites, such as folding sites or enzymatic active sites, can be more constrained than disordered segments of proteins [1]. Considering genes as units, there are variable degrees of constraints leading to different evolutionary rates acting on different genes. Evolutionary rate of genes has been extensively studied, being related to several factors - not necessarily concurrent - such as gene expression level [2], gene essentiality [3], gene duplication [4], connectivity of the gene products [5], and gene age [6,7].

It is possible to describe the cellular metabolism by a graph or network, where gene or gene products are represented by nodes and their associations, by links. From the point of view of gene networks, genetic modifications might affect both links (interactions among gene products) and nodes (gene products). Modifications on genes structure, such as single mutation, deletions, or insertions can modify the interactions between the mutated gene product and its network partners (*e.g. *proteins participating in the same pathway), altering links of their network. Events as gene duplication and horizontal gene transfer modify the gene network by inserting nodes. In addition, network nodes can be deleted by gene loss events [8]. Similarly to genes, different gene networks might be subject to different constraints being more or less tolerant to changes and likewise presenting different levels of genetic plasticity - the ability of a functional gene or gene network to tolerate alterations in its components or structure [9].

Plasticity is an elusive property, in the sense it cannot be directly measured and it is always required a subjacent model to design a proper measure. Different artificial model networks have been proposed to define plasticity measures, bringing interesting conclusions on the possible functioning of biological networks [10,11]. In addition, *in silico *techniques have shown good power of prediction for metabolic networks in unicellular organisms [12,13]. In complex multicellular organisms, however, there is paucity of data. In effect, determining the plasticity of a given gene network is far from a straightforward task also due to the incomplete knowledge about the relationships among gene-products as well as about their behavior in different environmental conditions [14]. Regarding genes, a possible manner to experimentally investigate genetic plasticity is by using deletion analysis and different projects have developed and organized gene deletion information for different model organisms [15,16]. In this case, robustness against gene deletion may be interpreted as a tolerance against alterations on the network (node deletion), implying a correlation with plasticity. Deletion information is relatively well established for unicellular organisms such as yeast; for mammals, however, it involves more complicated and expensive techniques and the information is somewhat incomplete, even for model organisms.

A relevant problem one faces when defining a plasticity measure has to do with time scales. Here we consider time scales long enough to allow for speciation. For these time scales there is consensus that, for example, the nucleotide excision repair (NER) system is highly conserved: both the set of genes and the biochemical reactions they participate in are fairly similar in every extant eukaryote on Earth. Although this set of genes appeared very early in evolution, they have not been often deleted in descendent species and they have not suffered many duplications. Accordingly, each DNA repair genes has an ortholog in almost all species, without many paralogs [9]. Following this reasoning, we can infer that conserved, non-plastic genes belong to families spread over all eukaryotes with few paralogs. On the other hand, one could expect that ancient, plastic genes would have suffered deletions, and duplications in some species, but not in others, throughout evolutionary times. The consequence for their ortholog groups would be i) not having orthologs in many species, and ii) when a given species has a gene in those groups, they will also present many paralogous genes.

The crescent sea of data generated by genome sequencing projects has provided raw material to investigate the evolutionary relationships among genes from different species. The analysis of large scale data about the distribution of gene families (*i.e. *genes possessing the same common ancestor gene - an orthologous group [17]) across non-exclusively closely related species can provide insights about how plastic or how conserved a given orthologous group has been throughout its evolutionary history. In some extent, this *evolutionary plasticity *of an orthologous group might bring a perspective on the genetic plasticity of their orthologous genes. The idea is to estimate for each group of orthologs in eukaryotes the number of genes and how they are distributed among the species. From this information, properly processed, one can characterize their evolutionary history. For this measure to yield information, it must discriminate different orthologous groups. As shown in what follows, this is possible, since a considerable number of gene families has components spread in virtually all eukaryotes, whereas a great number of orthologous groups is restricted to some specific lineages [6]. Accordingly, the distribution analysis of a gene family in a species group brings valuable information about how conserved and how old that gene family is [7]. A common way to evaluate the breadth and the depth of a gene family distribution is based in looking for gene presence and absence in an evolutionary tree [18-20]. An alternative way to evaluate the distribution of an orthologous group consists in using the Shannon information theory [21] to determine the diversity (*H_α_*) of its distribution in a species group [9]. This methodology is able to discriminate orthologous groups presenting patchy phylogenetic distributions - including lineage specific gene families - from broad distributed orthologous groups.

Molecular mechanisms such as gene duplication, exon shuffling, transposable elements, gene fusion and fission, and horizontal gene transfer have been related to development of new genes [22]. Among them, gene duplication has been discussed to be one of the most important events in genome evolution by providing the prime source of genetic material in which evolutionary forces can act generating novelty [23,24]. Duplication events occur randomly and duplicated genes can address different fates: (*i*) they can be selectively preserved, mainly by bringing an adaptive advantage; (*ii*) they can be selectively eliminated by bringing an adaptive disadvantage; and (*iii*) they can remain unoccupied, drifting in evolutionary process, eventually being eliminated or, more rarely, evolving to develop another biological function [25]. It is noticeable some orthologous groups possess one-to-one relationships, while there are gene families composed by a great number of paralogs [26]. The reason why some duplicated genes are fixed while others are eliminated has been extensively discussed; however, the mechanisms driving the destiny of the new-born duplicated genes remain controversial [25,27-29]. The Neo-Functionalization (NEO-F) and the Escape from Adaptive Conflict (EAC) are among of the most important theories about the fixation of duplicated genes. NEO-F represents the first idea of evolution by gene duplication and suggests that once duplicated, one of the gene copies turns free to acquire a new function in the course of the accumulation of neutral mutations, while another copy preserves the original biological function. EAC suggests that a pleiotropic gene performing more than one function - where each function could not be independently improved - will be beneficed by a duplication event where each gene copy is then free to specialize in each different function former performed by a single gene. A third theory is represented by sub-functionalization, where degenerating mutations happens in both duplicated copies that subdivides gene function between the duplicated genes. Consequently, both altered copies are preserved by selection since any individual former gene is able to entirely perform their biological function (for review, see [29]). A useful method to identify the importance of duplication events in the evolutionary history of an orthologous group is given by the ratio between the number of components present in the orthologous group and the number of organisms containing items from this orthologous group.

In a previous paper, we analyzed the distribution and the duplicability of a set of 142 orthologous groups extracted from STRING database http://string.embl.de/ to investigate the evolutionary origin of human apoptosis and genome stability gene network [9]. Here, we extended the analysis to all Eukaryotic Clusters of Orthologous Groups (KOG) available in STRING. Our goal here is to evaluate the evolutionary plasticity and conservation of an orthologous group according to the distribution of their components (*i.e. *orthologous and paralogous proteins). For each KOG present in STRING database, we calculate the diversity and abundance of their components across 55 fully sequenced eukaryotic genomes and suggest an equation to determine the evolutionary plasticity taking into account both diversity and abundance. To further KOG plasticity analysis, we estimate the evolutionary distance average among all proteins which take part in the same orthologous group from a sample of the KOGs present in STRING database. As a result, we found a strong correlation between the evolutionary distance average and the evolutionary plasticity index proposed. Additionally, we evaluate the evolutionary plasticity of mouse and yeast genes associated with lethality when knocked-out. We found low evolutionary plasticity in *Saccharomyces cerevisiae *genes associated with inviability and *Mus musculus *genes associated with early lethality. At the end, we plot the evolutionary plasticity value in different gene networks from yeast and human to identify their more and less evolutionary plastic areas as well as their more and less evolutionary conserved areas.

## Results

### Genes distribution within Orthologous Groups

**Figure 1:**
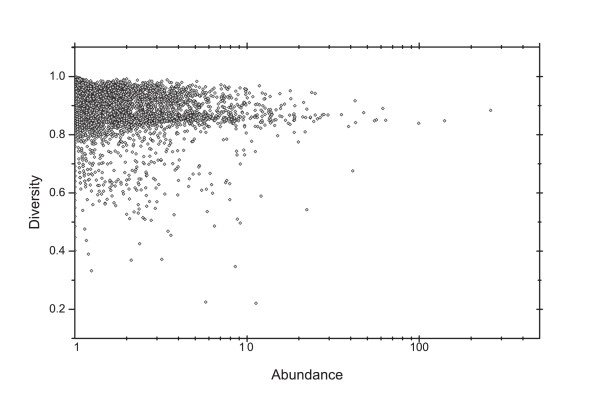
**Diversity (*H_α_*) and abundance (*D_α_*) distribution**. Each KOG present in STRING database was plot according to *H_α_*(y axis) and *D_α_*(x axis) values.

To assess the distribution of genes within each KOG we evaluated their diversity (*H_α_*) and abundance (*D_α_*) as described in *Methods *section. *H_α _*provides the distribution of a given orthologous group across a species group. High diversity indicates an equalized distribution of KOG components (*i.e. *orthologous and paralogous proteins) among the species evaluated. On the contrary, low diversity suggests a non-homogenous distribution. For a KOG to present maximal diversity their components are present in all species, meaning that this KOG ancestral gene arrived early in evolution, in the last common ancestor of all considered organisms - in our case, in the origin of eukaryotes or before. Furthermore, besides this ancestral appearing early in evolution, for its descendants to be found in all assessed genomes, deletion episodes cannot have happened very often. *D_α _*is defined as the average of number of proteins belonging to the same KOG, present in each organism. In general, high abundance denotes many duplication episodes in the evolutionary history of an orthologous group. Figure 1 shows the distribution according to *H_α _*and *D_α _*of all KOGs (4850 KOGs in total) present in STRING.

Note that there is a range of distribution, where *H_α _*of the majority of the KOGs is around 0.8 to 1, while *D_α _*is concentrated from 1 to 10. However, there are KOGs that show *H_α _*values lower than 0.8 as well as KOGs that present *D_α _*values higher than 10.

### Evolutionary Plasticity Index

Low values of *D_α _*combined with high values for *H_α _*indicates low plastic orthologous group, since it is present in many species, with few components, indicating it suffered few modifications (*i.e. *few duplication and deletion episodes) during eukaryotic evolution. Based on this, we have defined the evolutionary plastic index, *EPI*, to define how plastic a given orthologous group is, as follows:(1)

**Figure 2:**
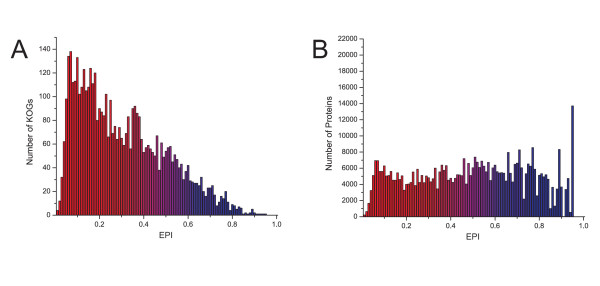
**Evolutionary Plasticity Index (*EPI*) distribution**. All KOGs present in STRING database were grouped in 100 categories according to *EPI *(A). All proteins present in KOG dataset were grouped in 100 categories according *EPI *(B).

Note that 0≤ *H_α_*≤1 and *D_α_*≥1. As a result, 0≤ *EPI *≤1. Figure 2A shows the distribution of all KOGs present in STRING organized in 100 groups according to *EPI*. Once identified the *EPI *of a given orthologous group, this information can be transferred to the proteins that compose this orthologous group (Figure 2B). The distribution of KOGs has its maximum displaced to low plasticity (Figure 2A); however, the distribution of proteins is roughly uniform (Figure 2B). This means that those KOGS with low plasticity present a lower number of proteins, strongly indicating a negative correlation between *EPI *and number of components (for further discussions, see Additional file 1, section 1.2).

### Evolutionary Distance versus *EPI*

Genes can differ in their evolutionary rates. Genes under purifying selection evolve slower compared to genes under Darwinian selection [30]. In this sense, analyzing the amino acid differences among gene products from the same orthologous group might give us an alternative plasticity evaluation of a gene family. We compared the amino acid sequences, all against all, for a sample of KOGs present in STRING using Poisson correction method [31,32] as described in *Methods *section. This method analyzes the differences in amino acid sequences and provides an evolutionary distance between two proteins. We used the average of all distances among proteins of the same KOG to take the evolutionary distance average of each KOG evaluated. Note that we did not evaluate synonymous substitution since the analysis was performed utilizing amino acid sequences. Therefore, every observed difference corresponds to non-synonymous substitutions.

**Figure 3:**
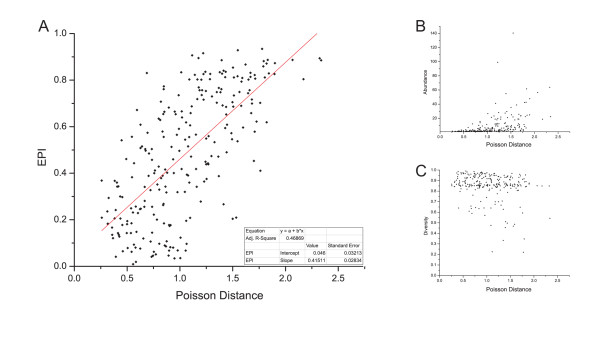
**Evolutionary distance average versus *EPI***. 5% of the KOGs present in STRING database were sorted. The evolutionary distance among all proteins of each KOG evaluated was calculated and the evolutionary distance average (Poisson Distance) was obtained. Poisson Distance was plotted against *EPI *(A), abundance (B) and diversity (C) of each KOG evaluated. Red line indicates the linear regression fitting curve and the box shows the curve proprieties.

Figure 3A shows a strong correlation (Pearson correction 0.68621, two-tailed test *p *< 0.0001) between *EPI *and evolutionary distance of the evaluated KOGs. KOGs that possess high *EPI *present high evolutionary distance among their gene products as well as KOGs identified as having low *EPI *possess proteins more similar to each other. According to Figure 3A, the components of a KOG presenting low *EPI *are more similar among each other, comparing to components of a KOG presenting high *EPI*. No correlation was identified when plotting evolutionary distance versus *D_α _*(Figure 2B), *H_α _*(Figure 2C), number of species, and number of proteins (see Additional file 1, Supplementary Figure S6).

### Functional Plasticity Analysis

**Figure 4:**
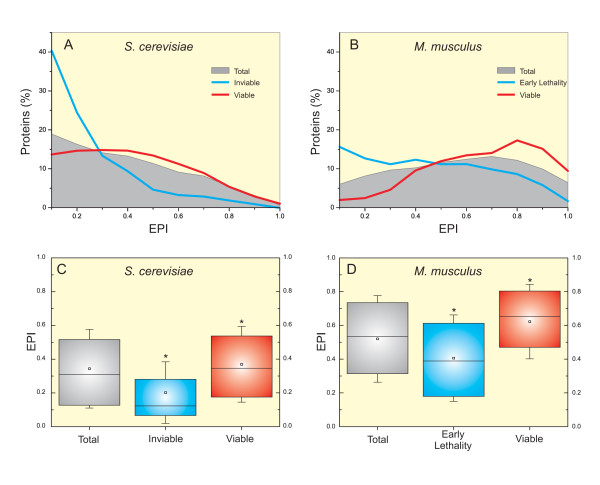
***EPI *distribution of target genes related with lethality when knocked-out**. The percentage of *S. cerevisiae *(A) and *M. musculus *(B) genes presenting different *EPI *values is show. The grey landscape represents the *EPI *distribution of all genes from each species. Blue lines represent the *EPI *distribution of *S. cerevisiae *genes associated with inviable phenotype when knocked-out (A) and *M. musculus *target genes associated with early lethality (B). Red lines represent the *EPI *distribution of target genes associated with viable phenotypes (A and B). Boxes represent *EPI *distribution of the different gene categories (total, inviable, and viable) from *S. cerevisiae *(C) and (total, early lethality, and viable) from *M. musculus *(D). The edges of the boxes indicate the upper and lower quartiles. The line at the center of each box indicates the median, and the whiskers represent the standard deviation. * indicates different from total group (*p *< 0.0001).

To verify correlation of EPI with previous estimates of genetic plasticity, we assessed knock-out data from *Saccharomyces cerevisiae *and *Mus musculus*, and looked for genes related with lethality. We considered two criteria to identify genes involved with lethality: (*i*) *S. cerevisiae *genes which confer inviability when knocked-out and (*ii*) *M. musculus *target genes which cause early lethality (*i.e. *lethality before placentation). Additionally, we considered as viable *S*. *cerevisiae *genes annotated as "viable" in SGD as well as *M. musculus *genes annotated as "no abnormal phenotype detected" without any phenotype annotation associated with lethality in MGI (to further discussion, please see *Supplementary material*, section 1.3). Figure 4 shows the distribution of proteins from *S. cerevisiae *(Figure 4A) and *M. musculus *(Figure 4B) according to *EPI*. The grey landscape represents the *EPI *distribution of all proteins of *S. cerevisiae *(Figure 4A) and *M. musculus *(Figure 4B) present in KOG dataset. Yeast proteins present a distribution concentrated in low *EPI*, while mouse proteins present a more uniform *EPI *distribution (to further discussion, please see *Supplementary material*, section 1.4). The *EPI *distribution of proteins codified by genes involved with lethality when knocked-out have their maxima displaced to low *EPI *in both yeast and mouse (blue lines in Figures 4A and 4B, respectively). The opposite can be observed when considering proteins codified by genes associated to viable phenotype when knocked-out (red lines in Figures 4A and 4B). Figure 4C shows that mean *EPI *of inviable group is significantly lower comparing to mean *EPI *from all *S. cerevisiae *proteins present in KOG dataset. In the same way, the early lethality group has mean *EPI *significantly lower as compared to the totality of *M. musculus *proteins found in KOG dataset (Figure 4D). Additionally, mean *EPI *of viable groups are significantly higher when compared to respective total groups in both *S. cerevisiae *and *M. musculus *(Figure 4C and 4D, respectively).

### Evolutionary Plasticity Index of biological networks

**Figure 5:**
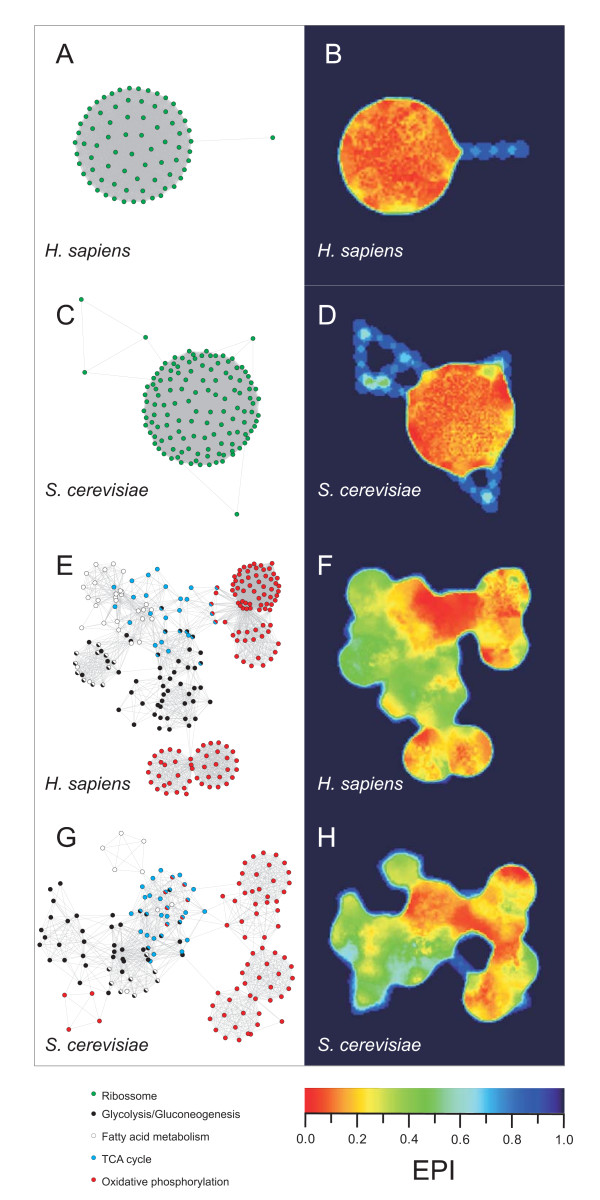
**Gene networks *EPI***. *EPI *projection onto different graph representation of gene networks from *S. cerevisiae *and *H. sapiens *are shown, with the respective network topologies. The nodes represent genes and the links represent protein-protein interaction of gene products (A, C, E and F). The color landscape indicates the *EPI *(B, D, F, and H). The nodes were colored according to the pathways they belong (A, C, E and F). Nodes with more than one color belong to more than one pathway evaluated (E and G). To complete list of genes, please see Additional file 1 (*Supplementary Figures *S7, S8, and S9), Additional file 5 (*H. sapiens *genes) and Additional file 6 (*S. cerevisiae *genes).

Cell functions are performed by functional modules [10,33] and gene network co-evolution has been proposed as an important evolutionary driving force agent [34]. In the same way, a network composed by proteins that take part in ancient and conserved KOGs can be regarded as conserved. To analyze the evolutionary plasticity of functional biological networks, we constructed the network of different pathways present in KEGG database http://www.genome.jp/kegg/ using protein interaction information from STRING (to further information, see *Methods *section). After network construction, we plotted the plasticity information of the network components (*i.e. *the *EPI *of the orthologous group of each gene from the network) onto network topology. Figure 5 shows a graph representation of ribosome network from human (Figure 5A) and yeast (Figure 5C). Ribosome network is formed by a single highly connected module in both, human and yeast, and both networks present low evolutionary plasticity in their components (Figures 5B and 5D). Figures 5E and 5G show a graph representation for networks from several energetic pathways from human and yeast. Each network comprises components from glycolysis/gluconeogenesis metabolism, fatty acid metabolism, tricarboxylic acid (TCA) cycle, and oxidative phosphorylation. Differently from ribosome network, which is composed by one module, energetic metabolism network possesses several interconnected modules. As we can see in Figures 5F and 5H, the region comprising TCA cycle presents the lowest evolutionary plasticity in both human and yeast. Oxidative phosphorylation presents low, even though not the lowest, evolutionary plasticity and both, glycolysis/gluconeogenesis metabolism and fatty acid metabolism, present the highest evolutionary plasticity of human and yeast energetic metabolism network. Complete graph representation of the networks with gene symbols are available in Additional file 1 (Supplementary Figures S7, S8, and S9).

## Discussion

Genetic plasticity estimative can be useful to different fields such as genetic diseases and evolution. For example, plasticity of a gene or a gene network can help finding components involved in pathology development as well as indicating possible therapeutical targets. Also, evolutionary novelty will probably appear on genome change-tolerant portions. The tolerance to modifications can be measured by directly modifying a gene structure or by estimating the gene variation in a population. Besides gene deletion experiments (a possible way of changing gene network structure), the presence of single-nucleotide polymorphism (SNP) (a way of estimating gene variation in a population) would be possible alternatives to evaluate genetic plasticity. However, a single nucleotide mutation may or not lead to a functional modification, depending on the site it occurs, leading to misevaluation of genetic plasticity. Copy number polymorphism (CNP) might work better in plasticity evaluation, mainly regarding entire deletions and duplication. In *Drosophila melanogaster*, for instance, around 8% of genes are at least partially duplicated and 2% are at least partially deleted, showing CNP as a common phenomenon and, consequently, an interesting target for genetic plasticity evaluation [35]. Genomic information has been largely used to predict biological function, from gene/protein function to entire gene/protein network architecture [14]. Co-inherence has been used to predict functional interaction between proteins [36] and computational techniques such as network alignment has been used to identify conserved pathways, manly in closely related organisms [37]. However, the evolutionary plasticity of orthologous groups has never been systematically analyzed.

Here, we have presented a large scale data analysis concerning the distribution of gene families across eukaryotes to identify conserved and plastic orthologous groups. It is noticeable the differences in orthologous groups distribution among eukaryotic genomes and those differences certainly hold biological information. The presence of a KOG component restricted to few eukaryotes indicate at least two possibilities: (*i*) the ancestral gene of this orthologous group arrived late in evolution and its orthologs are only observed in more recent taxa or (*ii*) the ancestral gene of this orthologous group arrived early, but its orthologs were lost in some of the taxa. Independently of the reason why a given orthologous group shows a patchy distribution among eukaryotes, it is clear that these orthologs are not required by all organisms. Conversely, a gene family widely found in eukaryotes plays an important role in virtually all organisms of this domain. Widely distributed genes have been described as being subject to stronger purifying selection as compared to young and less broadly distributed genes [6,7,18]. One hypothesis to explain these observations suggests that novel genes present an initial high evolutionary rate phase. At the end of this phase, there is a decrease in evolutionary rate due to an increased functional constraint [6]. Recent works in *D. melanogaster *have shown an adaptive evolution of young genes and an increased purifying selection as genes become older, corroborating that hypothesis [38,39]. Therefore, genes belonging to essential ancient gene networks, which optimized their roles early in evolution, are expected to present high conserved components across a species tree as well as few drastic modifications in the course of their evolution. On the contrary, genes which arrived late in evolution - or even in ancient non-essential gene networks - might present a patchy distribution among eukaryotes.

Other important feature concerning orthologous groups is represented by gene duplication. Why some genes possess several paralogs whereas other genes maintain one-to-one orthology relationships? Despite gene duplication occurring randomly, some genes are prone to fix a duplication event while other genes avoid duplication. The fixation of a duplication event is commonly associated with function improvement in new-born duplicated copies. A very good example is given by Jones and Begun in their study involving three independent events of evolution of chimeric fusion genes in *D. melanogaster*. All three studied genes are derived *Adh *and all three genes experienced a rapid evolution on the beginning of their history, followed by a slower adaptive evolution. Additionally, the authors have observed an intriguing similarity in the pattern of evolution including temporal, spatial, and types of amino acid changes in these proteins [39]. Those data strongly suggest that the parent-protein characteristics might determine the path a possible copy will experience, including whether or not it will be fixed or eliminated. According to EAC theory, genes exercising more than one function (*i.e. *genes presenting functional plasticity) are prone to fix a possible duplication event [28,40].

*EPI *is based on drastic changes in the history of orthologous groups such as gene duplication and gene deletion. However, a gene may experience different degrees of changes. A gene highly tolerant to mutations will accumulate alterations in its nucleotide sequence on the course of its history. On the contrary, a gene lowly tolerant to mutations will present few nucleotide alterations in its evolutionary history. A complementary, independent measure of the plasticity of an orthologous group is then given by the similarity among the sequences of their proteins. Low evolutionary distances indicate that the proteins present very similar amino acid sequences. Consequently, they suffered few modifications as compared to those proteins presenting high evolutionary distance. According to our results, *EPI *is correlated to the evolutionary distance measure, suggesting that genes widely distributed among eukaryotes and possessing few paralogs are subject to purifying selection, reinforcing the idea that they are conserved, low plastic genes. A recent work involving gene families in primates has shown an interesting relationship among family size conservation, evolutionary rates and gene essentiality. According to the authors, genes within size conserved families present lower evolutionary rate and a higher proportion of essential genes compared to genes within non size conserved families from human, chimpanzee and rhesus [41]. Those results suggest that our observation concerning duplicability, diminished evolutionary rate, and increased essentiality can also be observed by analyzing gene families in closely related organisms.

The idea is not new that essential genes are subjected to stronger selective constraints and, consequently, evolve slower than nonessential genes [42]. In this sense, evolutionary plasticity could be the reflex of genetic plasticity. According to our results, genes associated with lethality are significantly more related to low plastic orthologous groups than genes associated with no abnormal phenotype in both *S. cerevisiae *and *M. musculus*. Therefore, the evolutionary history of a gene, *i.e. *the distribution of their orthologs among different organisms, might bring information about the relevance of their role. However, some less common exceptions may occur. It may happen that some new duplicated genes evolve to perform essential functions, as represented by essential genes with high *EPI*. Chen and collaborators have shown new genes that rapidly became essential in *D. melanogaster*, exercising crucial roles mainly in intermediary or late stages of development [38]. However, a wide distributed gene without duplication and deletion episodes probably exercise important biological role, suggesting that *EPI *may have more acuity to determine low plastic genes than to high plastic genes.

Our hypothesis that the evolutionary plasticity of an orthologous group can be an indicative of genetic plasticity of genes within that orthologous group has been applied to ribosome and energetic metabolism gene networks, showing interesting results. Ribosomes are known as ancient molecular fossils that have arrived before the LCA of all living organisms [43]. As it has been shown here, ribosome gene networks of both *S. cerevisiae *and *H. sapiens *present very low *EPI*. The entangled network topology indicates an intricate relationship among the partners of this very ancient low plastic gene network. On the other hand, central metabolism has been described as highly variable among different prokaryotes [44,45]. Here, we have found fatty acid metabolism and glycolysis/gluconeogenesis as the highest plastic portion in central metabolism. Despite glycolytic pathway might have arrived early in evolution, its components are not conserved across the species and glycolysis has been described as a high plastic and versatile pathway [46]. Contrasting to glycolysis, TCA cycle represents the lowest *EPI *portion of the energetic metabolism network. Among the few works that have investigated the evolution of TCA cycle in eukaryotes, a recent paper has shown evolutionary similarity between mitochondria from *S. cerevisiae *and *Rickettsia prowazekii *in topological analyses based on network alignment and motif identification [47]. In the same work, the authors have described the mitochondria network as highly clustered around the TCA cycle. *R. prowazekii *is a mitochondria-related alpha-proteobacteria [48] and TCA cycle pathway seems to be closely related among eukaryotes and its ancestor prokaryote. Those assumptions agree with the results shown here, suggesting TCA cycle as low plastic and highly conserved among the eukaryotes. Despite the results shown here cannot be generalized to all biological networks, it opens a perspective on developing an extensive research concerning *EPI *and networks properties, such as node connectivity and clustering coefficient, as well as network centrality.

In the last decades, the advances in modern genomics have provided a powerful framework in the evolutionary research field. The availability of an enormous amount of completely sequenced genomes, including a great range of organisms, has provided new insights in evolutionary relationships involving genes, pathways, and species. *EPI *consists in a simple useful method that brings valuable complement in evolutionary studies and provides insights in other research fields such as pathology research and drug design. Clearly, the species set utilized in the orthologous group formation is essential to its diversity, abundance, and consequently to *EPI *determination. We avoid using the entire COG database due to its unequal distribution concerning the three domain of life (*i.e. *532 *bacteria*, 43 *archaea *and 55 *eukarya*). *EPI *can be applied to any species group to identify the evolutionary plasticity of gene families in related species. However, the researcher must take care with the evolutionary relationship among the species used in *EPI *determination to avoid biased results. Many evolutionary questions, such as the exact factors determining gene and gene networks evolvability, are still unsolved. Despite our work does not clarify how and why the modifications of some gene networks are constrict on the course of evolution, *EPI *represents one step in evolutionary relationship understanding by identifying which gene families have been more or less stable on the course of evolution.

## Conclusions

Our results suggest that the distribution of gene families brings valuable information on how plastic and how conserved a gene family is. It is possible to discriminate among conserved and plastic orthologous groups by evaluating their abundance and diversity. In addition, the evolutionary plasticity, measured according to orthologous group distribution as shown here, is coherent with other plasticity measures such as constriction in amino acid sequence modifications throughout evolution and essentiality in mouse and yeast. Finally, the evolutionary plasticity index measured according to abundance and diversity of gene families is consistent with the knowledge about the evolutionary conservation of ribosome gene network as well as the evolutionary plasticity of energetic metabolism gene network.

## Methods

### Data selection

Several databases offer tools in order to identify gene families. Each database utilizes its specific algorithm to find homology relationships according to specific purposes, such as to search orthologous genes/proteins along species or to search groups of genes/proteins which present the same last common ancestor (*i.e. *orthologous groups). However, the general strategy used by almost all database is to compare nucleotide sequences among different species [49-51] (to further discussion see Additional file 1, section 1.1). COG (Cluster of Orthologous Groups) database http://www.ncbi.nlm.nih.gov/COG presents a useful approach to identify orthologous groups. In COGs construction algorithm, all proteins encoded by the complete genomes analyzed are compared and for each protein, the best hit (BeT) in each different genome is detected. To name it as a cluster, it is necessary to form a triangle including BeT in at least three different organisms. Each COG represents a gene/protein family, including both orthologs and paralogs from different genomes, which have evolved from the same ancestral gene through a series of speciation and duplication events [52]. Besides COGs, which include eukaryotic and prokaryotic proteins, the database provides a tool involving only eukaryotic proteins. KOG (Eukaryotic Clusters of Orthologous Group) utilizes the same algorithm to find orthologous groups; however, it only works with eukaryotic genomes [53]. STRING database string-db.org has amplified the COG orthology information by creating more groups and adding extra species, totalizing 630 fully sequenced organisms with 55 eukaryotes among them [49]. Here, orthologous groups were accessed through STRING database version 8.2 stringdb.org [49], in download section. Only eukaryotic orthologous groups (KOG) were evaluated, resulting in a total of 4,850 KOGs. This dataset comprises 481,421 proteins distributed among 55 eukaryotes.

### Distribution of orthologous groups

An orthologous group corresponds to a set of genes belonging to different species, which have a common gene ancestor. To obtain a quantitative expression of the proteins distribution for each KOG (*i.e. *distribution of the items of a given KOG), we used Shannon Information Theory [9,21] defined as follows. Consider *n *as the number of selected KOGs, each one representing an orthologous group. Each KOG is labeled by α (α = 1,...,*n*) and has *N_α _*items (orthologous and paralogous genes), distributed among *M *possible organisms. Consequently, for a given KOG we can define *s*(*i*,α) as the number of items of a given organism *i*, (*i *= 1,..., *M_α_*), whose sum for a given α adds up to *N_α_*. The probability *p*(i,α) that, among the *N_α _*items of the α-KOG, a KOG randomly chosen, belongs to the organism *i *is written as(1a)

such that . The normalized Shannon information function *H_α _*is defined as(2)

where we have divided by ln(*M*) in order to normalize the quantities, guaranteeing that 0≤ *H_α_*≤1. Observe that if there is one gene per organism, *N_α _*= *M, p*(*i,α*) = 1/*M*, and *H_α _*= 1. In fact, *H_α _*reflects the spread of the distribution *s*(*i*,*α*), *i.e*., it measures the diversity that exists in the α-th KOG. *H_α _*near 0 indicates poor diversity, while a *H_α _*close to 1 suggests high diversity. The abundance *D_α _*of a given KOG was measured by obtaining the ratio between the number of items (orthologous and paralogous proteins) present in the KOG and the number of organisms containing items from this KOG. *D_α _*vary from 1 to virtually infinite (despite the higher abundance found here was around 260) and represents the average of orthologous and paralogs per species for a given KOG. The diversity and abundance was conducted using the software GenPlast. GenPlast have been designed by our research group to perform the plasticity analysis presented in this paper. The software has been developed in the java platform, is under an open source license, and is freely available at http://lief.if.ufrgs.br/pub/biosoftwares/genplast.

### Molecular evolutionary analysis

Molecular evolutionary analysis was conducted using MEGA version 4 [31]. 5% of the KOGs present in STRING (243 KOGs) was sorted according to the *EPI *and aligned amino acid sequences of all proteins comprising each KOG was obtained from STRING database string-db.org [49]. FASTA sequences were converted in MEGA format by the software. The number of amino acid substitutions per site between sequences was analyzed by the software set in "protein sequences". All results were based on the pairwise analysis sequences. Analyses were conducted using the Poisson correction method in MEGA4 [31,32]. All positions containing alignment gaps and missing data were eliminated only in pairwise sequence comparisons (Pairwise deletion option). The Poisson Distance average of all proteins contained in each KOG evaluated was also obtained using MEGA4. To complete list of sorted KOGs, please see Additional file 2 (*Supplementary Table S4*).

### Lethality Evaluation

*Saccharomyces cerevisiae *data was obtained from *Saccharomyces *Genome Database http://www.yeastgenome.org[16]. Genes associated to inviability when knocked-out was obtained using the SGD advanced search with step 1 (select chromosomal feature) set in "ORF" and step 2 (narrow results), box phenotype properties, set in "Inviable". Genes associated to viable phenotype when knocked-out was obtained following the same procedure, except by shift "inviable" by "viable" in phenotype properties box. The complete list of *S. cerevisiae *genes with phenotype annotations used here is available in Additional file 3 (*Supplementary Table *S5). *Mus musculus *data was obtained from Mouse Genome Informatics http://www.informatics.jax.org[54] in download area, file "Genotypes and Mammalian Phenotype Annotations (tab-delimited)". The following phenotype annotations were considered together to form the group "early lethality": embryonic lethality before implantation [MP:0006204], embryonic lethality at implantation [MP:0008527], embryonic lethality between implantation and placentation [MP:0009850], embryonic lethality before somite formation [MP:0006205], and embryonic lethality before turning of embryo [MP:0006206]. Genotypes with more than one target allele were discarded. Genes possessing the phenotype annotation "no abnormal phenotype detected [MP:0002169]" were considered to form the "viable" group. MGI BioMart version 0.7 http://biomart.informatics.jax.org was utilizing to find knock-out target genes. Genes combining MP:0002169 and any other phenotype annotation associated with lethality (MP:0005374, MP:0002081, MP:0002058, MP:0002080, MP:0008762, MP:0008527, MP:0006204, MP:0009850, MP:0006205, MP:0006206, MP:0006207, MP:0006208, MP:0005373, MP:0008569, and MP:0002082) were discarded. After that, all resultant genes codifying proteins preset in KOG dataset was utilized. The complete list of *M. musculus *genes with phenotype annotations used here is available in Additional file 4 (*Supplementary Table *S6).

### Network Plasticity

The protein-protein interaction networks were generated using information from KEGG http://www.genome.jp/kegg/[51] and STRING string-db.org [49] databases in two steps. First, the gene set of human and yeast pathways evaluated was obtained from KEGG. Only human genes identified in HUGO Gene Nomenclature Committee http://www.genenames.org/[55] and yeast genes identified in *Saccharomyces *Genome Database http://www.yeastgenome.org[56] were used. Second, protein interaction was obtained using STRING database with input options "databases", "experiments", and 0.700 confidence level. STRING integrates different curated public databases containing information on direct and indirect functional protein-protein associations. Network was constructed including only interacting genes/proteins and results from the search were saved and further handled in Medusa software [57]. Evolutionary plasticity of each network was determined in two steps. First, the *EPI *of each protein from the network was determined according to the *EPI *of the KOG to which the protein takes part. Second, the evolutionary plasticity data was plot onto the network using the software ViaComplex [58] to construct a landscape representation. The complete list of *H. sapiens *genes used to construct the networks is available in Additional file 5 (*Supplementary Tables S7*) and the complete list of *S. cerevisiae *genes used to construct the networks is available in Additional file 6 (*Supplementary table 8*).

## Competing interests

The authors declare that they have no competing interests.

## Authors' contributions

RJSD designed the study, performed the analysis, discussed the results, and write the manuscript. MAAC designed the study and discussed the results. JLRF performed the analysis and discussed the results. LHTS performed the analysis. RMCA designed the study and critically reviewed the manuscript. JCFM designed the study and critically reviewed the manuscript. All authors have read and approved the final manuscript.

## Reviewers' comments

### Reviewer 1

*Professor Manyuan Long, Department of Ecology and Evolution The University of Chicago*.

This reviewer provided no comments for publication.

### Reviewer 2

*Hiroyuki Toh*,

The authors evaluated the evolutionary plasticity based on the diversity and the abundance of the orthlogous genes. The authors found that the plasticity is associated with the inviability of yeast and the early lethality of mouse. The approach is interesting. However, I found several problems in the manuscript. Following is the list for possible amendment.

#### Major: problems

(1) The authors defined "genetic plasticity" as the ability of a functional gene network to tolerate the alterations in its components or structures (p. 3 line 7 Background). In page 7 (Results, Evolutionary Plasticity Index), the authors defined "evolutionary plasticity" as formula (1), which is calculated with the diversity and the abundance of orthologous genes.

(1-1) Is the term "genetic plasticity" equivalent with the term "evolutionary plasticity"?

***Authors' response: **Actually, these two concepts are different. Genetic plasticity is a gene property, while evolutionary plasticity is an orthologous group property. Genetic plasticity, as described on the manuscript, corresponds to the gene (or gene network) capacity to tolerate changes and the evolutionary plasticity, defined by Eq.1, is the record of changes a given gene family have experienced through its evolutionary history. We rewrite a substantial part of the introduction to clear both concepts. We also added additional discussion to elucidate the differences, as well as the relationships, between both*.

(1-2) If the two terms are used to indicate the same thing, it is not clear why the value calculated with the diversity and the abundance of the orthologous genes can indicate the ability of a gene network, since the formula (1) is given for a component of a gene network.

***Authors' response: **As mentioned above, genetic plasticity and evolutionary plasticity are not the same thing. However, we propose a relationship between both. Starting to the point that genes do not work alone in an organism, the capacity of a gene to tolerate changes will certainly be influenced by their gene network. Additional discussions were added to the manuscript involving the relationship among the gene plasticity and the gene network plasticity*.

(2) about the term "paralog" used in the manuscript.

The authors used the eukaryotic clusters of orthologous group (KOG) in this study. To define the diversity and the abundance in p. 17 - 18 (Methods, Distribution of orthologous groups), the authors used not only orthologs but also paralogs. The description iseems to be confusing for the readers who are not so familiar with the genome science, since the term paralog" used in the manuscript is not the general one, I think that the authors wanted to indicate "co-ortholog" by "paralog". So, I think that a KOG does not include the distant paralogs. I recommend the authors to check the usage of the terms. Unless only close paralogs or co-orthologs are considered for the calculation of formula (1) in p. 18, the diversity loses the meanings. If the authors wanted to include the distant paralogs for the calculation, the consideration of the taxonomic bias may be required. Likewise, the definition of the abundance may be too naïve. Let's consider two cases with two species. In the first case, only one species has 99 paralogs, whereas the other has one orthologs. Da is calculated as (1+99)/2 = 50 in this case. In the other case, the first species has 50 paralogs and the other has remaining 50 copies. In this case, Da is calculated as (50 + 50)/2 = 50. That is the same values are obtained for the two cases. The first case may reflect a trend for the species specific gene amplification, whereas the second case may suggest the duplicability of the orthoologs. I think that the taxonomic bias should be taken into account for the calculation of Da.

***Authors' response: **In fact, there are some controversies involving the terms ortholog and paralog. Other terms such as co-ortholog, inparalog, outparalog, pseudoortholog, pseudoparalog, etc, can be added to the debate. The strict description of each of those terms is not the point here. Our point is to discriminate among orthologous groups possessing one ortholog per species analyzed and orthologous groups possessing many orthologs (or co-orthologs) per species analyzed. Additionally, we analyze the distribution of ortholog among species to discriminate broadly distributed orthologous groups from poorly distributed orthologous group. As we do not include taxonomic relationships in the analysis, we analyze the set of species as a whole, independently of the distance among them. A fungi-specific orthologous group, for instance, will present low diversity. In the same way, a primate specific orthologous group also will present low diversity. In contrast, an orthologous group that has components equally present in all species evaluated will have high diversity. In what concerns KOG database, it intends to identify all eukaryotic genes which evolve from the same ancestral gene*.

*We agree with the reviewer in their comment relative to abundance. Abundance cannot be used without diversity to evolutionary plasticity inference, as shown in *Figure 3B*. This is the reason why we use the abundance combined to diversity. Examining the suggested example:*

*Case 1: one species has 99 paralogs, whereas the other has one ortholog. In this case, the abundance is 50 and the diversity is 0.080793136. Accordingly, EPI is 0.988574125*.

*Case 2: one species has 50 paralogs, whereas the other has 50 copies. The abundance is 50, exactly equal the case 1. The diversity, however, is 1. In this second case, EPI is 0.858578644. As shown, different orthologous groups presenting equal abundance but different diversity will have different EPI*.

#### Minor problems

(1) The authors pointed out the importance of neo-functionalization after gene duplication. However, the authors did not mention sub-functionalization. I think that the dubfunctionalization is also related to the evolutionary plasticity. Why did the authors neglect the subfunctionalization.

***Authors' response: **The theories discussed on the manuscript (i.e. neo-functionalization and EAC) are two important examples among many others about gene duplication theory. Since the reviewer judged important to mention sub-functionalization, a comment about that theory has been added on the manuscript*.

(2) The authors used "aminoacid" instead of amino acid in the manuscript. I think that "amino acid" is ordinarily used.

***Authors' response: **It has been modified*.

(3) p. 19 -20 (Methods, Fitness Evaluation

The term "fitness" is used for different meanings from that in the evolutionary biology and the population genetics. I recommend the authors to use different term to express "fitness" in their manuscript.

**Authors' response: **We have replaced "fitness" by "genes involved with lethality when knocked-out"

(4) p.10 (Results, Functional Plasticity Analysis) naïve idea on evolution

It may be my misunderstanding, but some descriptions in p.10 seem to be naïve as an evolutionary statement.

(4-1),p. 10 line 1 "increase in complexity is a hallmark of evolution.

Evolutionary biologists do not consider so. Degeneration and neural change are also important to consider the evolution.

***Authors' response: **We agree with the reviewer. In fact, there are examples of evolution by diminishing the complexity. The meaning intended with the sentence is related to life as a whole. Since first life forms have arrived, crescent levels of complexity can be observed in life organization. Despite simple organisms still represent the majority of the life forms, the complex relationships between different organisms and the environment is noticeable. To avoid misunderstanding we have changed "evolution" by "life" on the manuscript*.

(4-2) p. 10 lines 2 - 4

However, impairment in biological networks whose have arrived early in evolution (i.e. before multicellularity) might lead to early developmental lethality.

(5-1) whose ----➩ which

***Authors' response: **Alteration has been done*.

(5-2) There is no rationale or citation for this statement, but the authors seemed to follow the recaptulation theory by Heckel, which is still in debate. The authors should provide the rationale of this statement.

***Authors' response: **We agree with the reviewer and removed the sentence from the main manuscript. We added an extra section in the Supplementary Material, discussing lethality in multicellular organism. We provide the rationale of that statement on this new section*.

### Reviewer 3

*Sebastien Halary*,

### Referee 3 - S. Halary

This study proposes an index called Evolutionary Plasticity Index (EPI) to assess the "genetic plasticity" of genes. This index is defined as a function of the abundance (number of genes) and distribution (diversity of organisms having these genes) within the homologous genes family a gene belongs to. EPI was calculated for 4850 KOGs and compared for 243 of them with their Poisson distance average of all proteins they contain. Then, EPI utility was illustrated by comparing the 'plasticity' of lethal against non-lethal genes of *S. cerevisiae *and *M. musculus*, and the plasticity of genes involved in interactions/metabolic networks. EPI seems to be a simple tool to assess the diversity of a gene in eukaryotes, and then to be useful to characterize the paralogs richness of a homologous genes family. Nevertheless, this paper does not provide satisfactory arguments to justify the use of EPI rather than the other existing tools used up till now to estimate the diversity within a homologous family. This is mainly because the results are not discussed in sufficient depth. The authors propose to investigate relationships between EPI and lethality or topological position of the protein in a network, but did not compare their results with previous studies on the same subjects, whereas it could be useful to assess the power of their approach. To improve the manuscript, I would recommend that the authors provide concrete examples for which their index outperforms existing indices, or for which the tool is more straightforward. Also, the discussion can be improved by being more specific about optimal condition for this tool and/or by specifying novel applications.

From an editorial point of view, this paper is very long, mainly because of repetitions (without taking account of the 6 supplementary files). Many paragraphs are not placed in the suitable chapter. The quality of language could sometimes be improved upon as well. Overall, this results in a confusing article.

***Authors' response: **We thank the reviewer for the extensive revision he had provided. We followed his suggestions as possible, improving substantially the paper. We also identify some misunderstanding and have worked on improve the clearness of the discussions*.

*We agree with the reviewer and made efforts to make the paper as short as possible. We removed some peripheral discussions from the main manuscript to the supplementary files. We also have replaced many paragraphs in order to clear the reading. Language has been revised*.

In my opinion, this article cannot be published before major editing and some revisions. I have some questions about the methods and the results, which I hope could be useful to improve the manuscript:

-How were the 5% of KOGs chosen for the comparison EPI/evolutionary distance? Why 5%?

***Authors' response: **Data analyzed here involves a total of 481,421 proteins distributed among 4850 KOGs. It is a large, however finite, population. To better estimate the relationship among EPI and evolutionary distance, we take a large sample (i.e. n/N>0.05. In our case N = 4850. Accordingly, n would be > 242.5). A sample larger than 5% would be unnecessary and would substantially delay the paper*.

-There is a correlation between EPI and 'evolutionary distance', but it would be quite dangerous to resume the second by the first. These values provides more complementary than comparable information. You can find 2 KOGs with the same EPI, and very different means of distance (Figure 3A). Anyway, the authors discuss neither, nor do they comment on the relevance of their index. Which methods already exist to assess diversity of genes within a homologous family? Why is your index better than others or what kind of supplementary information can it provide?

***Authors' response: **We completely agree with the reviewer. Evolutionary distance is complementary to EPI since both evaluate different classes of changes. While EPI identify entire gene alterations (i.e. duplication and deletion episodes), evolutionary plasticity evaluate the amino acid variation among the proteins. Since each measure evaluates different things, one cannot be explained exactly by a function of the other. Our results show that wide-distributed orthologous groups that have experienced few duplications and deletions episodes tend to have proteins more similar among each other (according to amino acid sequence), i.e. we found a coherent relationship between EPI and evolutionary distance, as shown by *Figure 3A. We have amplified the discussion about EPI and evolutionary distance relationship to clear it and to avoid misunderstanding.

*Our analysis does not attempt to replace any existing method and the point here is the possibility to evaluate a great amount of data and extract information from it. The relationship among orthologs distribution and the orthologous group plasticity cannot be neglected and the present manuscript is the first work concerned in systematizing this relationship. We also have improved the discussion on other works concerning in evaluate gene networks plasticity to clear the usefulness of our research*.

-The list of genomes in STRING DB (as I can read in the legend of Figure S5) is composed by 34 genomes from animals, 14 from fungi, 1 from plant and 6 from "protists" (belonging to 3 different kingdoms). First, for the figure S5A, if you choose to make the distinction between animals and fungi which are phylogenetically quite close. It could make sense to also make the distinction between the "protists" (mycetozoa, euglenozoa, alveolata and diplomonads) which are very distant from each other. Second, since there is just one plant in the dataset, you are not able to see the plant-specific KOGs and thus, you could underestimate the number of plant-specific paralogs and EPIs. Following the same reasoning, this study cannot be adapted to non-fungal unicellular organisms of the dataset. Actually, the dataset seems to be only suitable to assess animal and fungal protein diversity. What do you think about the possibility to adapt the set of genomes per study, to the organism of interest?

***Authors' response: **The figure S5 attempts to show the EPI differences comparing complex multicellular and unicellular/simple multicellular organisms. Our intention is to discuss the relationship among the organism complexity (i.e. multicellular, unicellular) and EPI. The phylogenetic relationship among the groups is not the point here. We removed the figure S5A since we judge figure S5B as sufficient to discussion. Additionally, we add a new section on supplementary material to better discuss the results concerning EPI in different organisms. Regarding the second point, we agree with the reviewer. Species set is not appropriated to obtain conclusions on specific taxonomic groups such as plants. This is the reason way we do not infer any conclusion based on specific taxonomic groups. On the contrary, we just evaluated if an orthologous group is wide-distributed or narrowly-distributed among the 55 eukaryotes analyzed. We think is a good idea to use EPI to evaluate subsets of organisms and thank the referee for the suggestions. We add on the manuscript a discussion about this possibility*.

-There are some "lethal proteins" with high EPI. Could you present one of these cases and discuss that?

***Authors' response: **We have added an example of lethality in novel proteins of D. melanogaster. Additionally, we extend the discussion (supplemental material) regarding EPI and lethality*.

-You present lethal/non-lethal proteins study and network plasticity as two different cases of application, but it is probable, at least for some proteins, that their "lethality status" is related to their centrality and/or connectivity in the interactions network.

***Authors' response: **We agree with and thank the reviewer for the suggestion. Indeed, many works have suggested an association among lethality and different networks properties, such as centrality and connectivity. Here, we found a connection between lethality and evolutionary plasticity, and a possible relationship among evolutionary plasticity, lethality, and networks properties may exist. One of ours perspectives is to perform a research involving evolutionary plasticity and networks properties*.

-You cite Li et al. 2006, which present the study of duplicability of genes in yeast. You could have cited also Chen et al 2010 (MBE) article which present a close investigation in humans. More precisely, I think you could have compared their results to yours to assess the efficiency and usefulness of your index before applying it to another question, even a simple one, to improve the quality of your discussion. You have focused your discussion exclusively on the importance of duplication in evolution, but you did not provide new evidence or hypotheses.

***Authors' response: **We thank the reviewer to suggest the very good paper of Chen and collaborators. We have used their results in our discussion about duplicability and evolutionary rate*.

- It is a good idea to use your index to study diversity within metabolic/interaction networks. However, even if the 4 shown examples are interesting, they can not constitute any evidence about the importance of low EPI proteins within networks in general. A more convincing approach would have been to make an exhaustive study of protein's EPIs in function of their network's node properties. Centrality and connectivity measures should be useful to identify the proteins that you need to investigate in the aim to discuss about EAC theory, for instance.

***Authors' response: **We completely agree with the reviewer. Our results must be evaluated as an example of EPI utilization. We have added a comment on discussion section to make it clear. As mentioned before, we plan to perform an extensive research involving evolutionary plasticity in a networks perspective*.

-The Figure 5 is very pretty, but it needs to be modified to improve the clarity of the results. First, (and at least) you must invert the both columns, since even in the text you began by describing the right one. Second, the resolution of the coloration in the left column is too low and it is often difficult to make the correlation between a node and its EPI. I propose to remove this column and to plot coloration directly on the network's nodes. For instance, Cytoscape allows to colorize a node and its outline in different colors. Furthermore, you don't provide a simple description of these networks in the legend and/or in Results: what are nodes, what are edges and what do the edges length mean?

**Authors' response:**

*First: the columns have been inverted*.

*Second: we think may be a good strategy coloring the nodes to identify EPI values of the genes. However, it is not our objective here. The software ViaComplex, used to produce the figure, work by projecting a landscape onto a network to identify the area of influence of a given property, such as transcription level, lethality, or evolutionary plasticity. The software takes in consideration the nodes and the links between nodes to project the information (here, to project EPI information). To access EPI of a specific gene of the presented networks the reader can check supplementary tables S7 and S8*.

*Third: the figure brings a graph representation of different networks and the information regarding nodes and edges are presented on the figure legend. Additional figures with gene symbols are shown on supplementary material*.

Please consider these detailed suggestions:

p.1: 2 semi-colons in the authors list.

***Authors' response: **The commas have been substituted by semicolons in the author list*.

p. 2: in the Background section of the Abstract: "duplicability (abundance) and distribution (diversity)". Is the abundance of genes in a COG (breadth of the COG) only a function of their duplicability? Can these 2 words be used as strictly synonyms.

***Authors' response: **Every molecular mechanism involved with the development of new genes might be related to the abundance of an orthologous group. Horizontal gene transfer, for instance, can increase the abundance of an orthologous group by adding extra gene copies in a given genome, increasing the orthologous group abundance as a whole. Nevertheless, such episodes are far from greatly relevant in abundance constitution, mainly in eukaryotic organisms, whereas gene duplication is admittedly the most important mechanism. In addition, the exact molecular mechanism involved in abundance (i.e. gene duplication, reverse transcription, etc.) is not the point here. To avoid misunderstanding, however, we changed the referred sentence on the abstract*.

p. 3: "Genetic plasticity may be understood...". Exact repetition of the first sentence of the abstract.

***Authors' response: **We have changed the sentence on the background section*.

p.3: «The analysis of a large scale data about the distribution of genes families (i.e orthologous group)». You did not survey families of orthologous genes *stricto sensu*, otherwise you should not have observed duplication events. Ortholog being a confusing term, especially when you use COGs from eggNOG database (which provides the db of STRING I think), it would be helpful to fix the definitions of homo/ortho/para-logous gene.

***Authors' response: **We agree with the reviewer in their concernment about orthologs. It has been extensively discussed and there is no consensus about the nomenclature. The evolutionary relationships among genes involve several possible mechanisms that turn difficult to determine if a couple of genes in different species (or sometimes in the same species) are orthologs, coorthologs, paralogs, inparalogs, outparalogs, pseudoorthologs, or pseudoparalogs among each other. Despite such different relationships indeed exist, in practice, however, the identification and classification of homology relationships remains very difficult, mainly to entire genomes comparisons involving several species. The concept of orthologous group is exactly projected to characterize a group of genes with a same common ancestor, which is the meaning intended here. To make it clear, we have added this concept on the manuscript as well as the citation of a very explicative review wrote by Professor Koonin. Regarding the origin of the dataset, eggNOG and KOG represent distinct projects. KOG is based on a robust manual expert annotation whereas eggNOG is automatically and computationally constructed. For reference, please check Muller et al Nucl Acids Res 2010, 38: D190-D195*.

Then, the sentence p.4 « It is noticeable some orthologous groups possess one-to-one relationships, while there are gene families composed by a great number of paralogs» could be replaced by «Then, some homologous gene families are only composed by orthologs, while others possess a great number of paralogs too.»

***Authors' response: **We think that to consider as orthologs all one-to-one relationship could be a mistake in some cases, according to discussed above. Let's examine the following example: There are two out-paralogs (gene A and gene A') in two related species (species x and species y). In this example, the gene A_x _(i.e. the gene A from the species x) is ortholog of A_y _(i.e. the gene A from the species y) and the gene A'_x _is ortholog of A'_y_. However, during the speciation process, the ortholog A has been deleted in a new species w (which possess only the gene A'_w_) and the ortholog A' has been deleted in another new species k (which possesses only the gene A_k_). Analyzing the species w and k, the genes A'_w _and A_k _are not orthologs among each other in spite of a one-to-one relationship involving the referred genes. Again, is very difficult to determine the exactly evolutionary relationship among genes. This is the reason way we prefer to use the orthologous group concept in our analysis*.

p.3: «from broad orthologous group» groups.

***Authors' response: **The alteration has been done*.

p. 3 to p. 4: «In a previous paper, we analysed [...] to the genes which codifying such proteins. » These lines must be displaced in the last paragraph of the introduction. Furthermore, the syntax is not correct in « the genes which codifying such proteins ».

***Authors' response: **The lines have been replaced and the last sentence has been removed*.

p. 6: «To assess the distribution of each KOGs», I would prefer «To assess the distribution of genes within each KOGs». In the same way, maybe you can change the title to make it more precise.

***Authors' response: **The alterations have been done*.

p. 7: «As mentioned above, a KOG presenting *Hα *and *Dα *[...] few duplications episodes.» This sentence can be removed.

***Authors' response: **The sentence has been removed*.

p. 7: «It is reasonable to think that a KOG with those [...] *Hα *indicates a high plastic orthologous group.» More Discussion or Introduction than Results.

***Authors' response: **The sentence has been modified*.

p. 7: «The distribution of KOGs is dislocated». Is "dislocated" the best term ?

***Authors' response: **The term has been replaced*.

p. 8: «Accordingly, a randomly chosen protein has [...] characteristic of an index (to further discussion, see Additional file 1, section 1.2).» Discussion

***Authors' response: **The sentence has been modified*.

p. 8: « Genes can differ according to evolutionary rates [...] plasticity evaluation of a gene family.» Discussion

***Authors' response: **I agree with the reviewer that the discussion section would be a good place to the pointed sentence. However, we prefer provide a short introduction to situate the reader on the issue that will be presented. Additionally, the maintenance of the sentence will not disturb the objective of the section*.

p. 8: «We compared the aminoacid sequences [...] those proteins presenting high evolutionary distance.» Methods

***Authors' response: **To the same reasons discussed above, we prefer to maintain a substantial part of the paragraph. The end of the paragraph, however, has been placed in the discussion section*.

p.8 to p. 9: «A gene highly tolerant to mutations [...]few nucleotide alterations in its evolutionary history.» Not Results.

***Authors' response: **The sentence has been placed in the discussion section*.

p. 9: «That result suggests that genes widely distributed [...] they are conserved low plastic genes.» «Those results reinforce [...] than *Dα *or *Hα *individually.» Discussion. Furthermore, EPI better than *Dα *or *Hα*, but is EPI better than 'evolutionnary distance' ???

***Authors' response: **First, the sentences have been placed in the discussion section. Second, we did not make that statement regarding EPI better than evolutionary distance. Evolutionary distance is a measure of the divergence of amino acid sequence among proteins, i.e. it evaluates changes in protein's structures. EPI works with other kind of changes: gene duplications and gene deletions. So, they are complementary measures*.

p. 9: « Starting to the point that low [...] fitness impact when knocked-out...» to simplify.

***Authors' response: **The paragraph has been rewritten*.

p.9: «*S. cerevisiae *information was obtained [...]Genome Informatics (MGI) [21].» Methods

***Authors' response: **The sentence has been removed*.

p. 9: « Is not new the idea that [...] annotation associated with lethality in MGI.» Introduction/Discussion/Methods...not Results.

***Authors' response: **A substantial part of the paragraph has been placed on discussion section*.

p.11: « To analyze the evolutionary plasticity [...] (*i.e. *the *EPI *of the orthologous group of each gene from the network) onto network topology. » Methods

***Authors' response: **We believe that a brief introduction is important for a better presentation of the results of *Figure 5.

p. 12: «The evaluation of the history of a gene or a gene network is fundamental to understand its evolutionary behavior.» Do you mean that we need to know the evolution of a gene to understand its evolution? I think this sentence is not useful. Discussion must be revised. To improve the clarity of the discussion, you can follow the same structure than the Results chapter.

***Authors' response: **The sentence has been removed. We substantially changed introduction and discussion sections*.

Methods must be simplified.

***Authors' response: **Methods section has been simplified as possible*.

## Supplementary Material

Additional file 1 **Supplementary Material**. Document containing supplementary results and discussion, including 23 figures and 3 tables. Click here for file

Additional file 2 **Supplementary table S4**. Table containing the orthologous groups sorted to evaluate the evolutionary distance among their proteins. Click here for file

Additional file 3 **Supplementary table S5**. Table containing *Saccharomyces cerevisiae *genes possessing phenotype annotations involved with inviability or viability when knocked-out. Click here for file

Additional file 4 **Supplementary table S6**. Table containing *Mus musculus *genes possessing phenotype annotations involved with "early lethality" or "no abnormal phenotype" when knocked-out. Click here for file

Additional file 5 **Supplementary table S7**. Table containing *Homo sapiens *genes used to construct the networks to illustrate different biochemical pathways. Click here for file

Additional file 6 **Supplementary table S8**. Table containing *Saccharomyces cerevisiae *genes used to construct the networks to illustrate different biochemical pathways. Click here for file
