# Supplementary material for: Evolutionary plasticity determination by orthologous groups distribution
Source: Biol Direct. 2011 May 17;6:22. doi: 10.1186/1745-6150-6-22 (PMC3117832; doi:10.1186/1745-6150-6-22)
Supplement: Additional file 1 — Supplementary Material. Document containing supplementary results and discussion, including 23 figures and 3 tables. [file 1745-6150-6-22-S1.PDF]

Evolutionary plasticity determination by orthologous groups distribution

Supplementary Material

Rodrigo JS Dalmolin[1§], Mauro AA Castro [1], José L Rybarczyk Filho[2], Luis HT Souza[1]; Rita MC de Almeida[2]; José CF Moreira[1].

[1]Department of Biochemistry, Institute of Basic Health Sciences, Federal University of Rio Grande do Sul, Rio Grande do Sul, Brazil.

[2]Department of Physics, Institute of Physics, Federal University of Rio Grande do Sul, Rio Grande do Sul, Brazil.

Correspondent Author – Rodrigo JS Dalmolin. Departamento de Bioquímica, ICBS-UFRGS. Rua Ramiro Barcelos, 2600, anexo. Laboratório 32. Phone +55 51 33085578, fax +55 51 33085540. E-mail: rodrigo.dalmolin@ufrgs.br

Contents

1. Supplementary results and discussion…..……….……………………………3

  1.1. Distribution of orthologous groups from different datasets…………….3

  1.2. EPI equation determination……………………………..…………….4

  1.3. Lethality information…………………………………………………….9

  1.4. *EPI* distribution in different species…………………………………….10

2. References………………………………………………………………………30

List of Supplementary Figures

Supplementary Figure S1. Diversity and abundance distribution of orthologous groups from different databases…………………..…..……………………………………...4

Supplementary Figure S2. Evolutionary Plasticity distribution according to different equations………..…………………………………………………………….5

Supplementary Figure S3. Evolutionary Distance Average versus Evolutionary Plasticity according to different equations……………………………...…………….6

Supplementary Figure S4. Distribution of target genes with different fitness impact according to Evolutionary Plasticity calculated using different equations………………8

Supplementary Figure S5. *EPI* distribution of different groups according to complexity…………………………………………………………………………...11

Supplementary Figure S6. Evolutionary Distance Average versus number of proteins and number of organisms………………………………………………………12

Supplementary Figure S7. Human and yeast ribosome gene networks…..………..…13

Supplementary Figure S8. *S. cerevisiae* energetic metabolism gene network…...…..14

Supplementary Figure S9. *H. sapiens* energetic metabolism gene network………..…15

Supplementary Figure S10 to S23. EPI distribution of different species…………….16

1. Supplementary results and discussion

1.1. Distribution of orthologous groups from different datasets

The three most important databases concerned in organizing orthologous groups are Inparanoid Database (http://inparanoid.sbc.su.se), KO (Kegg Orthology) Database [1] (http://www.genome.jp/kegg/ko.html), and COG (Cluster of Orhologous Groups) Database (http://www.ncbi.nlm.nih.gov/COG) [2]. Inparanoid is designed to find orthologs and in-paralogs between two species and to separate in-paralogs from out-paralogs, excluding out-paralogs in orthologous groups formation [3]. Therefore, it is inappropriate to use Inparanoid orthologous groups here, since our objective is to find all proteins that have the same ancestor gene. Both COG and KO work in gathering all paralogs and orthologs that possess the same ancestral gene in the same orthologous group. We avoid working with prokaryotic genomes (*i.e.* bacteria and archaea) due to their higher proportion of horizontal gene transfer events comparing to eukaryotes [4]. Accordingly, we have used KOG dataset to perform our investigation instead of using the entire COG database (including 55 eukaryotes and 575 prokaryotes) or KO database (including 149 eukaryotes and 1164 prokaryotes). KOG extracted from STRING Database (http://string.embl.de/) represents a curated dataset to identify protein families with the same ancestral gene [5]. Supplementary Figure S1 shows abundance (D_α) and diversity (H_α) distribution of all orthologous groups from three different datasets: COG, KOG, and KO. While KOG orthologous groups present a concentrated H_α distribution (around 0.8 to 1), both COG and KO presents a wide H_α distribution, probably due to high heterogenicity of the species (*i.e.* eukarya, archaea, and bacteria) that compose each dataset.

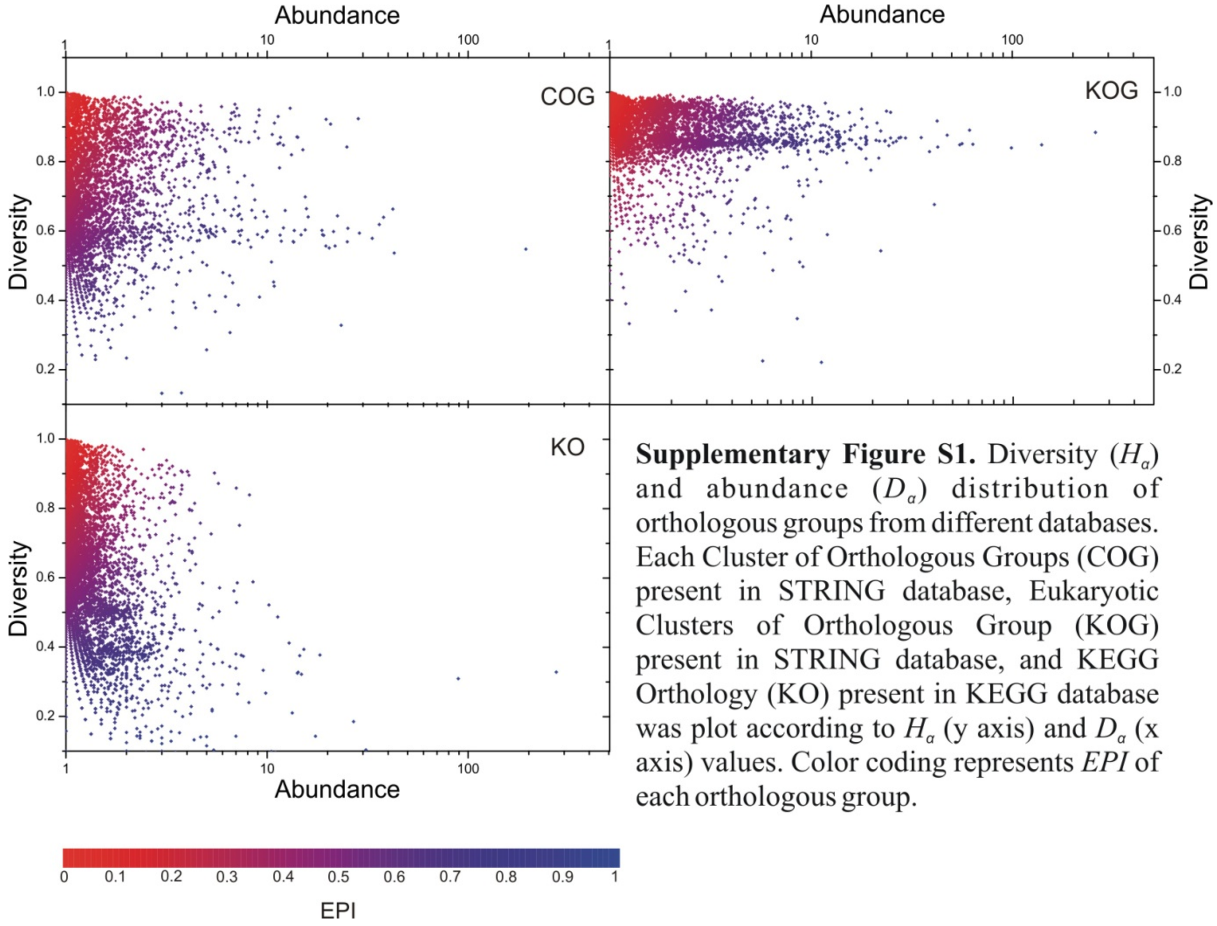

Supplementary Figure S1. Diversity (H_α) and abundance (D_α) distribution of orthologous groups from different databases. Each Cluster of Orthologous Groups (COG) present in STRING database, Eukaryotic Clusters of Orthologous Group (KOG) present in STRING database, and KEGG Orthology (KO) present in KEGG database was plot according to H_α (y axis) and D_α (x axis) values. Color coding represents *EPI* of each orthologous group.

1.2. *EPI* equation determination

According to what was discussed in the main text, evolutionary plasticity of an orthologous group is positively correlated to their D_α and negatively correlated to their H_α. The most intuitive way to produce an index comprising diversity and abundance together is the ratio between both. However, not necessarily the ratio between H_α and D_α will best represent the evolutionary plasticity of an orthologous group. Supplementary Figure S2 shows all KOGs present in STRING (Supplementary Figure S2.A and S2.C) and all proteins that compose those KOGs (Supplementary Figure S2.B and S2.D). KOGs and proteins were organized according to evolutionary plasticity as follows: equation (1) (Supplementary Figure S2.A and S2.B) and equation (2) (Supplementary Figure S2.C and S2.D).

$$Evolutionary\ Plasticity = 1 - \frac{H_\alpha}{D_\alpha} \tag{1}$$

$$Evolutionary\ Plasticity = 1 - \frac{H_\alpha}{\sqrt{D_\alpha}} \tag{2}$$

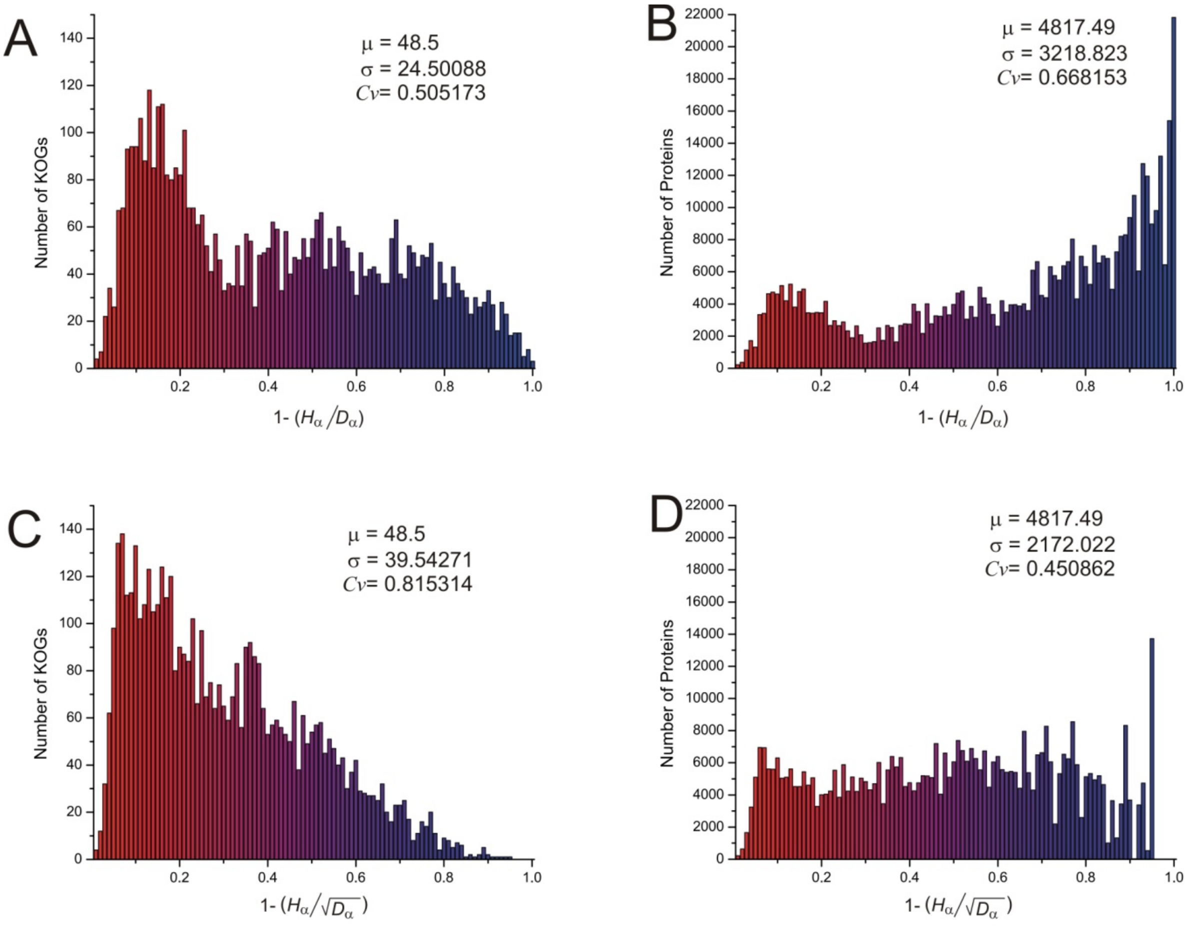

Supplementary Figure S2. Evolutionary Plasticity distribution according to different equations. All KOGs and proteins present in STRING database were grouped in 100 categories according to evolutionary plasticity calculated according to equation (1) (A and B, respectively) and equation (2) (C and D, respectively). Color coding is proportional to evolutionary plasticity. μ = mean, σ = standard deviation, and *Cv* = coefficient of variation.

The distribution of all proteins present in KOG dataset was dislocated to high plasticity when equation (1) was used to determine evolutionary plasticity (Supplemental Figure S2.B). Conversely, we observed an equalized protein distribution according to evolutionary plasticity when using equation (2) (Supplemental Figure S2.D). Thus, a protein randomly chosen among all species present in KOG dataset has similar probability to show low, median, or high evolutionary plasticity using equation (2) to determine evolutionary plasticity. Following those criteria, equation (2) has been elected to describe the Evolutionary Plasticity Index (*EPI*). To certify the competence of equation (2) comparing to equation (1), we repeated evolutionary distance analysis and functional plasticity analysis using both equations to determine evolutionary plasticity. Both analyses have been performed with the same methodology described in *Material and Methods* section (*Molecular Evolutionary Analysis* and *Fitness Evaluation*), except when changing equation (2) by equation (1) in *EPI* determination. Supplementary Figure S3 shows two correlation graphics between evolutionary distances among all proteins present in a same KOG and evolutionary plasticity of that KOG calculated by equation (2) (Supplementary Figure S3.A) and by equation (1) (Supplementary Figure S3.B).

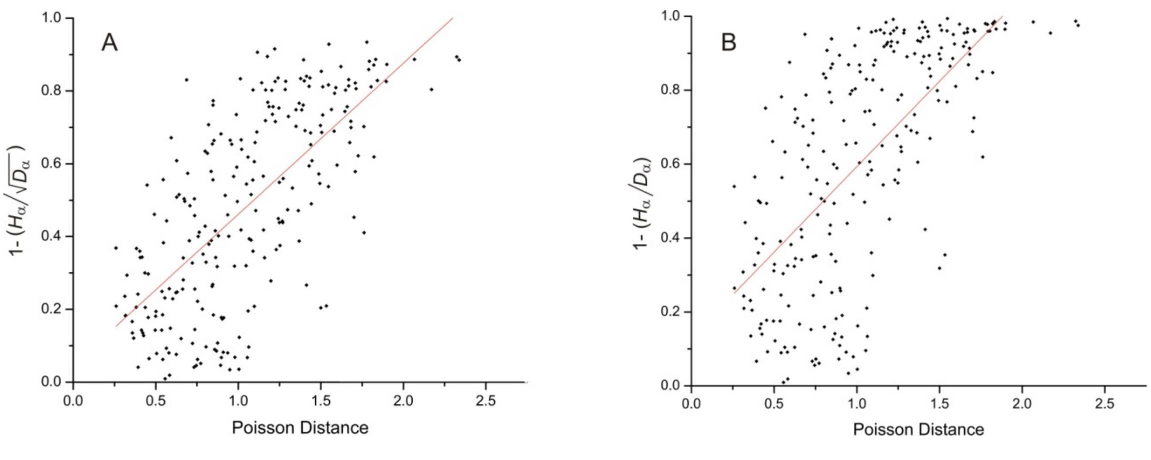

Supplementary Figure S3. Evolutionary Distance Average versus Evolutionary Plasticity according to different equations. 5% of the KOGs present in STRING database were sorted. The evolutionary distance among all proteins of each KOG evaluated was calculated and the evolutionary distance average (Poisson Distance) was obtained. Poisson Distance was plotted against evolutionary plasticity calculated according to equation (2) (A) and according to equation (1) B. Red lines indicates the linear regression fitting curve.

Supplementary Table S1 shows Pearson Correlation, as well as fitting curve properties, of both graphics. Despite both graphics have shown a correlation between evolutionary distance and evolutionary plasticity, the graphic generated using equation (2) (*i.e.* graphic A) presented higher Pearson Correlation. In addition, all linear regression fitting curve properties, such as intercept, slope, and residual sum squares, was more adequate in graphic A comparing to graphic B (Supplementary Table S1), reinforcing equation (2) utilization.

Supplementary Table S1. Linear regression fitting curve properties

	Graphic A		Graphic B	
Pearson Correlation	0.68621		0.66268	
Adj. R-Square	0.46869		0.43682	
Residual Sum of Squares	9.17866		12.93475	
	Value	Standard Error	Value	Standard Error
Intercept	0.046	0.03213	0.13076	0.03815
Slope	0.41511	0.02834	0.46222	0.03365

Supplementary Figure S4 shows the distribution of proteins from *S. cerevisiae* (Supplementary Figure S4.A and S4.C) and *M. musculus* (Supplementary Figure S4.B and S4.D) according to *EPI* calculated by equation (2) (Supplementary Figure S4.A and S4.B) and equation (1) (Supplementary Figure S4.C and S4.D). As shown in Supplementary Figure S4, the same phenomenon observed when using equation (2) to calculate evolutionary plasticity (as presented in *Results*, section *Functional Plasticity Analysis*) can be observed when using equation (1). In fact, an improvement in the differences of the means can be observed using equation (1) when compared to equation (2) (table S2 and table S3). However, except by differences in Z-value of *Saccharomyces cerevisiae* viable group, the differences are not outstanding.

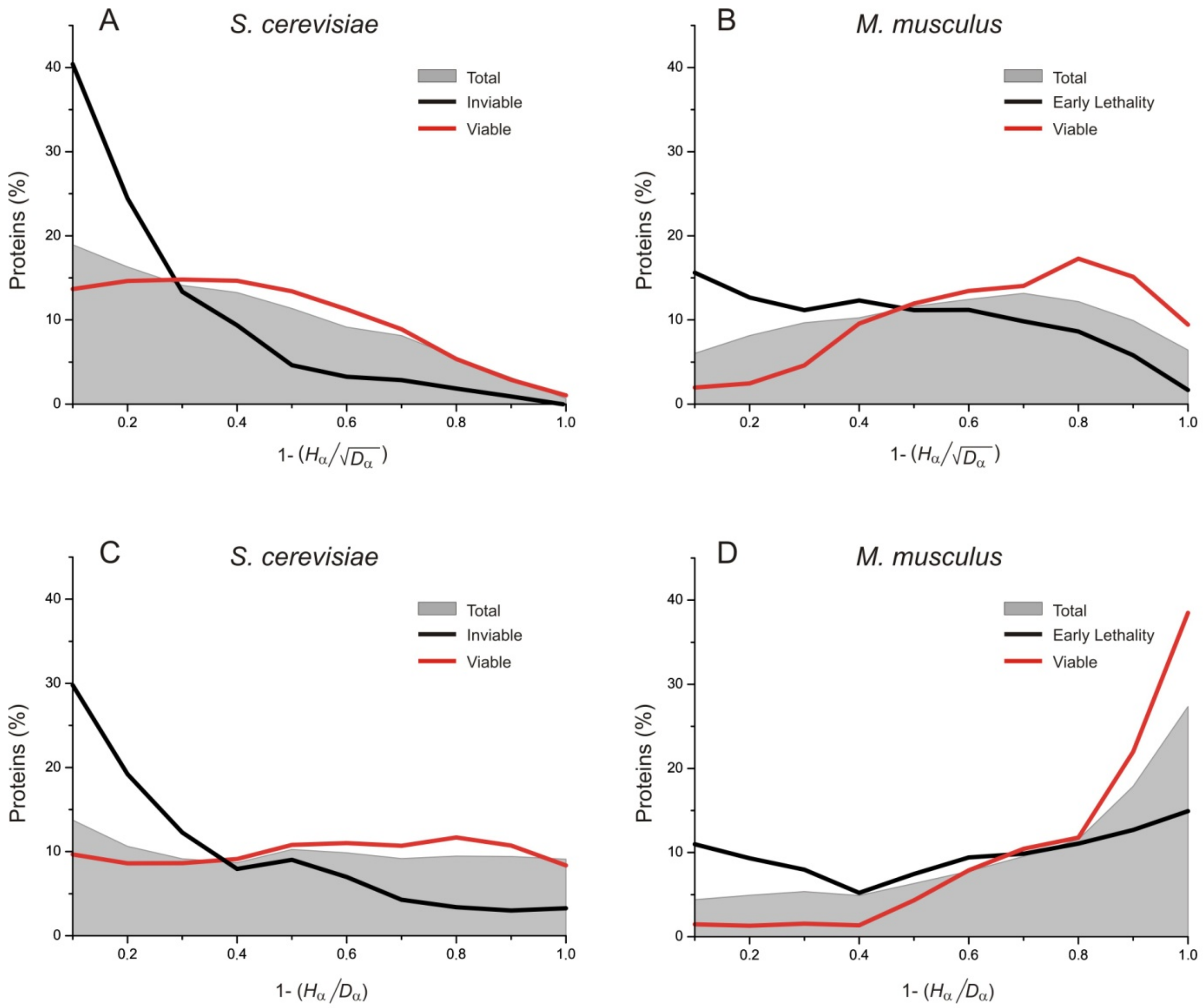

Supplementary Figure S4. Distribution of target genes with different fitness impact according to Evolutionary Plasticity calculated using different equations. The percentage of *S. cerevisiae* and *M. musculus* genes presenting different evolutionary plasticity values calculated using equation (2) (A and B) and equation (1) (C and D). The grey landscape represents the *EPI* distribution of all genes from each species. Black lines represent the *EPI* distribution of *S. cerevisiae* genes associated with inviable phenotype when knocked-out (A and C) and *M. musculus* target genes associated with early lethality (B and D). Red lines represent the *EPI* distribution of target genes associated with viable phenotypes (A, B, C, and D).

Supplementary Table S2. Descriptive statistics of *S. cerevisiae* genes.

	Number of Proteins	Mean	Standard Deviation	Standard Error	|Z-value|
Equation (1)					
Total	3998	0.47477327	0.292309	-	-
Inviable	891	0.29451211	0.2503	0.00979272	18.40767312
Viable	2792	0.51078711	0.2793	0.00553203	6.510059513
Equation (2)					
Total	3998	0.3430	0.2334	-	-
Inviable	891	0.2010	0.1832	0.0078	18.1569
Viable	2792	0.3690	0.2242	0.0044	5.8988

Supplementary Table S3. Descriptive statistics of *M. musculus* genes.

	Number of Proteins	Mean	Standard Deviation	Standard Error	|Z-value|
Equation (1)					
Total	14919	0.6726	0.2821	-	-
Early lethality	368	0.5474	0.3076	0.0147	8.5165
Viable	244	0.7858	0.2164	0.0181	6.2674
Equation (2)					
Total	14919	0.5199	0.2565	-	-
Early lethality	368	0.4061	0.2561	0.0134	8.5169
Viable	244	0.6226	0.2211	0.0164	6.2501

1.3. Lethality information

Several studies concerning essentiality and evolutionary parameters (*e.g.* duplicability and evolutionary rate) have been performed, hardly ever present apparent conflicting results [6-9]. However, care must be taking to compare unicellular and multicellular organisms according to lethality. Commonly, a mammalian gene is considered lethal when its deletion leads to organism death in any phase of development, in the first moments after birth, or even by causing infertility. Following those criteria, one will find essential genes which have arrived in different moments of evolution, turning difficult to trace a relationship among any evolutionary parameter and lethality in mammals. Increase in complexity is a hallmark of life [10] and the impairment of any organizational level can be lethal to complex organisms. However, impairment in biological systems which have arrived early in evolution (*i.e.* before multicellularity) might lead to early developmental lethality. In other words, a system such as DNA repair is important to unicellular organisms and a disruption in its homeostasis can be lethal to the cell, leading to early lethality. Systems involved in maintaining tissue homeostasis (*e.g.* apoptosis) may lead to lethality, however in earlier development stages compared to DNA repair, since a single cell con survive without apoptosis [11]. In a recent paper, Chen and colleagues have investigated the effect of young genes deletion in *D.*

melanogaster. They observed lethality associated with young genes mainly in middle or late stages of development. Indeed, no one deleted young gene lead to lethal phenotype at first or second larval instar [12]. Those results agree with the idea that young genes have less probability to lead to early lethality.

1.4. *EPI* distribution in different species

According to what was discussed in main text, simple organisms have a great proportion of low *EPI* genes comparing to complex organisms. Supplementary Figure S5 shows *EPI* distribution of all proteins present in KOG dataset from different taxonomic groups (complex multicellulars, fungi, and protista). There is a similar distribution between complex multicellular organisms, as well as between fungi and protista (Figure S5 and Figures S10 to S23). While the average *EPI* of proteins from complex multicellular organisms is around 0.5, average *EPI* of proteins from simple organisms is around 0.35. Supplementary Figure S5 considers average *EPI* from each species (*i.e.* average *EPI* from all proteins from a given species) from complex multicellular organisms (*i.e.* metazoa and plantae merged), fungi, and protista. While *EPI* from fungi and protista species do not significantly differed among each other, *EPI* from the species of both groups are significantly lower than *EPI* from complex multicellular species. Multicellular organisms possess genes that have appeared in different moments of evolution. For example, a great number of genes responsible to cellular homeostasis arrived before multicellularity advent (*e.g.* DNA repair genes [13]), whereas a great proportion of the genes involved in cell-cell communication arrived during multicellular evolution (*e.g.* TNF family [14]). However, unicellular organisms might possess higher proportion of ancient conserved genes when compared to mammals or other multicellular organisms.

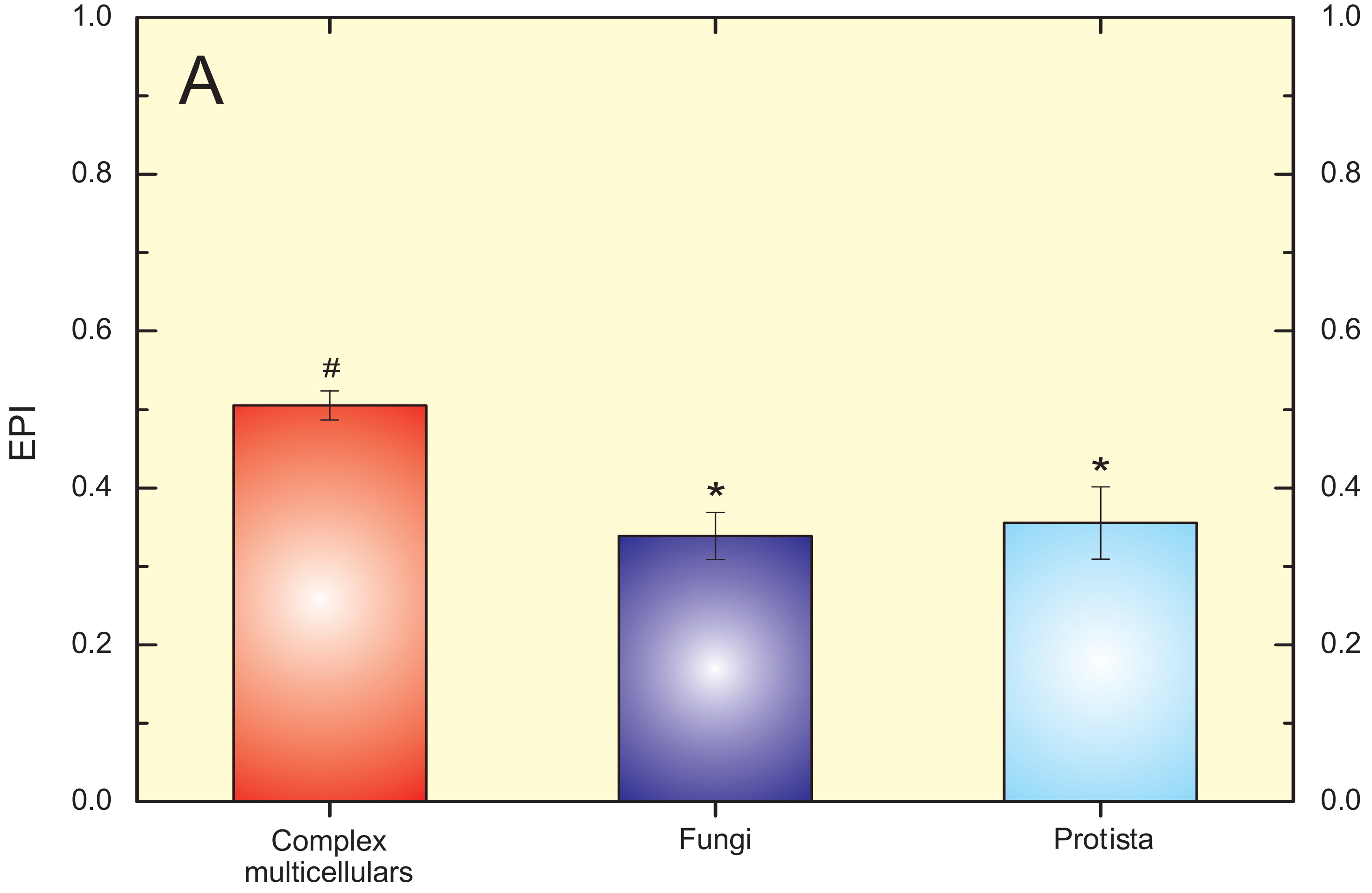

Supplementary Figure S5. *EPI* distribution of different groups according to complexity. Proteins of different groups were considered together to identify *EPI* distribution of the respective group. Complex multicellular organisms: *Homo sapiens*, *Pan troglodytes*, *Macaca mulatta*, *Otolemur garnettii*, *Tupaia belangeri*, *Mus musculus*, *Rattus norvegicus*, *Spermophilus tridecemlineatus*, *Cavia porcellus*, *Oryctolagus cuniculus*, *Canis lupus, Felis catus*, *Erinaceus europaeus*, *Sorex araneus*, *Bos Taurus*, *Myotis lucifugus*, *Loxodonta Africana*, *Echinops telfairi*, *Dasypus novemcinctus*, *Monodelphis domestica, Ornithorhynchus anatinus*, *Gallus gallus*, *Xenopus tropicalis*, *Oryzias latipes*, *Gasterosteus aculeatus*, *Takifugu rubripes*, *Tetraodon nigroviridis*, *Danio rerio*, *Ciona intestinalis*, *Ciona savignyi*, *Aedes aegypti*, *Anopheles gambiae*, *Drosophila melanogaster*, *Caenorhabditis elegans,* and *Arabidopsis thaliana*. Fungi organisms: *Kluyveromyces lactis*, *Eremothecium gossypii*, *Candida glabrata*, *Debaryomyces hansenii*, *Saccharomyces cerevisiae*, *Pichia stipitis*, *Yarrowia lipolytica*, *Neurospora crassa , Gibberella zeae*, *Aspergillus fumigatus*, *Schizosaccharomyces pombe*, *Ustilago maydis*, *Filobasidiella neoformans*, and *Encephalitozoon cuniculi*. Protista organisms: *Plasmodium falciparum, Cryptosporidium parvum, Leishmania infantum, Trypanosoma brucei, Dictyostelium discoideum, and Giardia lamblia.* Mean *EPI* of each organism were considered to evaluate the variation in *EPI* of each group. The whiskers represent the standard error. Asterisk (*) are equal among each other and different from number sign (#). $P<0.001$ ANOVA one-way, Bonferroni post-hoc.

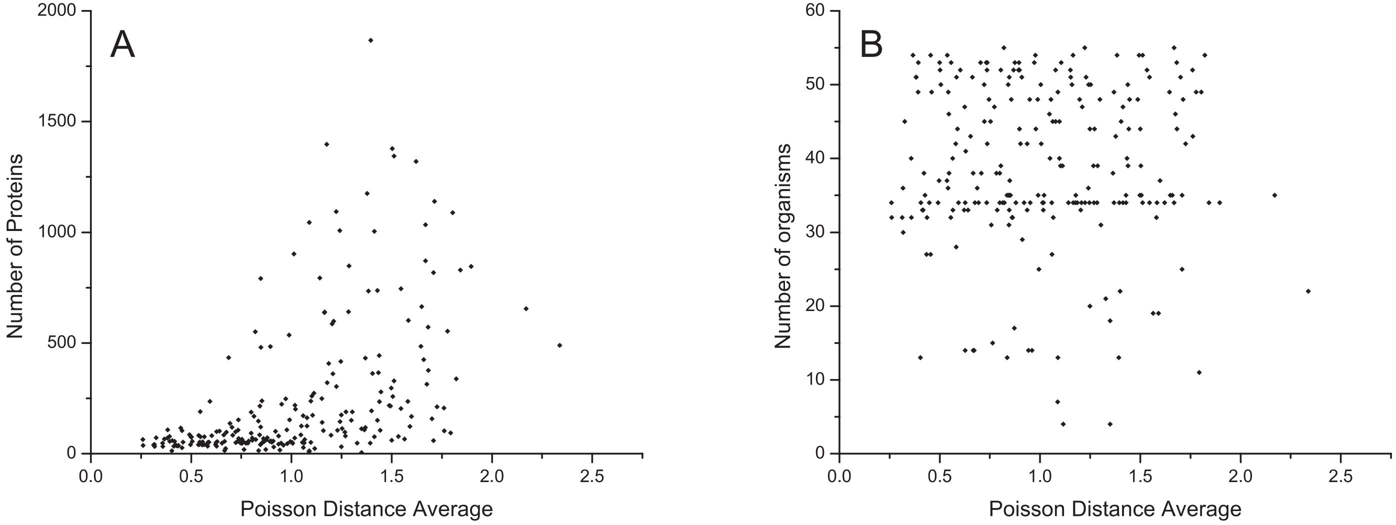

Supplementary Figure S6. Evolutionary Distance Average versus number of proteins and number of organisms. 5% of the KOGs present in STRING database were sorted. The evolutionary distance among all proteins of each KOG evaluated was calculated and the evolutionary distance average (Poisson Distance) was obtained. Poisson Distance was plotted against number of proteins (A) and number of organisms (B) of each KOG evaluated.

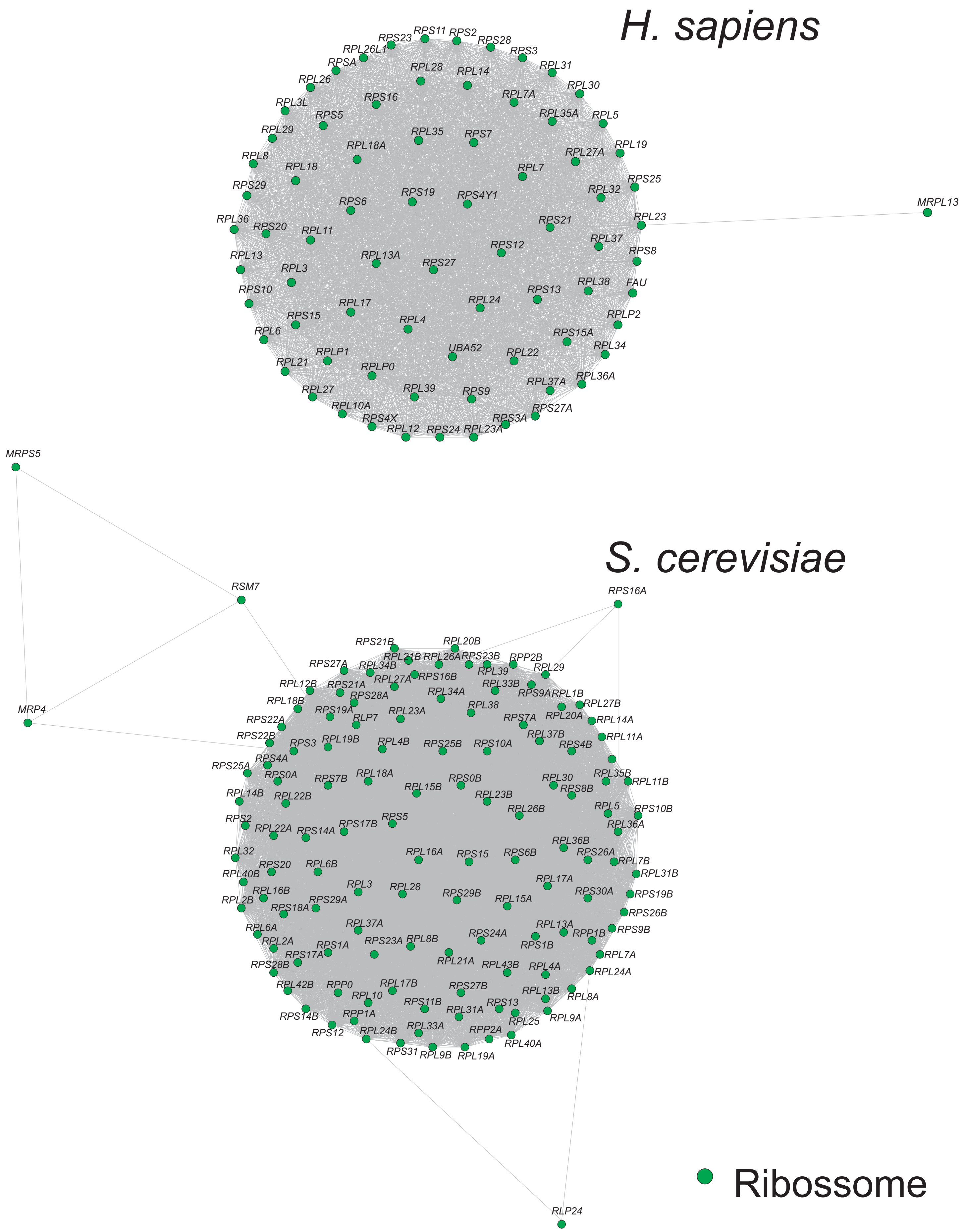

Supplementary Figure S7. Human and yeast ribosome gene networks. Ribosome protein-protein interaction networks of *H. sapiens* and *S cerevisiae* are shown. Nodes represent genes and the links represent protein-protein interaction of gene products.

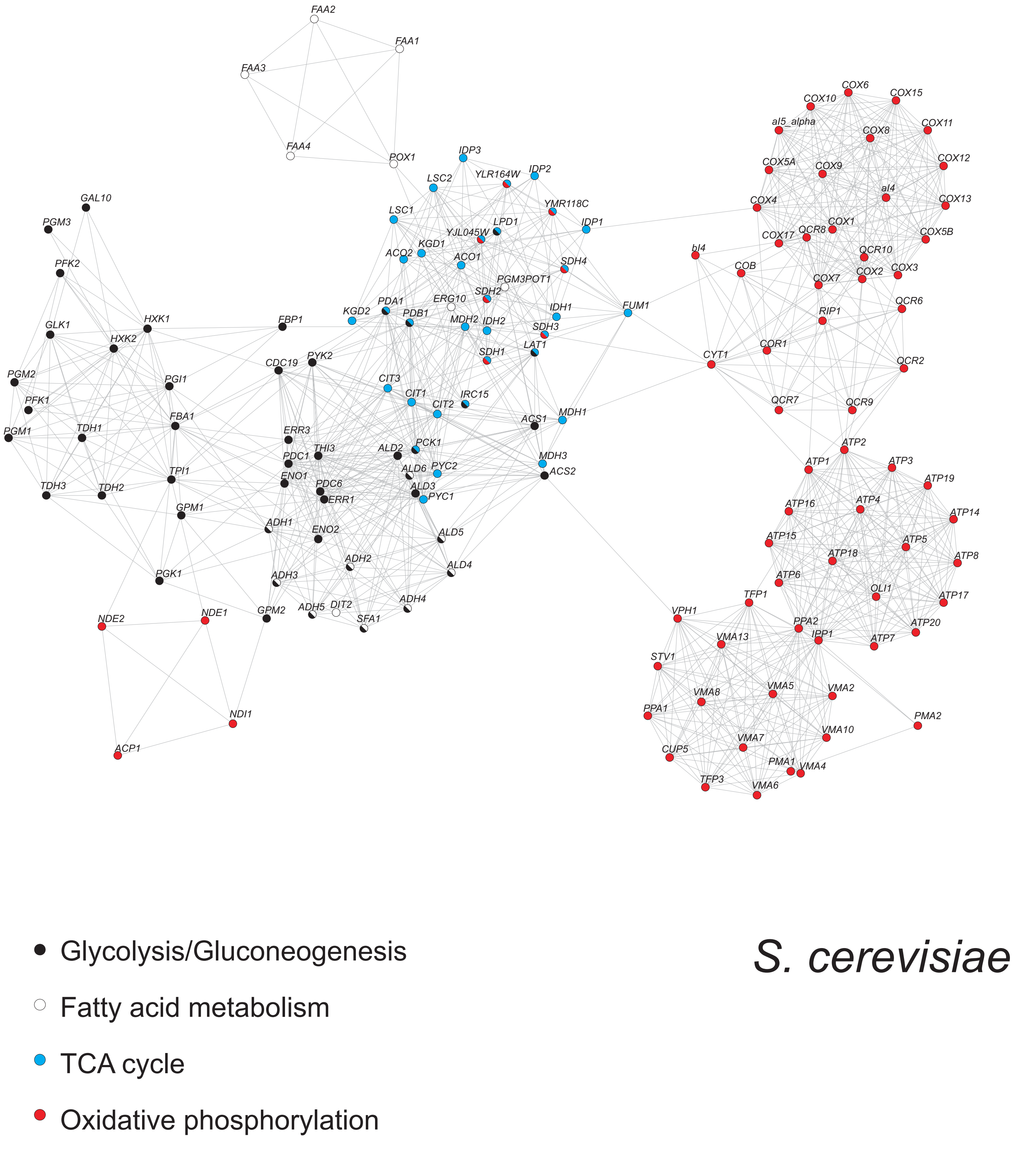

Supplementary Figure S8. *S. cerevisiae* energetic metabolism gene network. Energetic metabolism protein-protein interaction network of *S. cerevisiae* is shown. Nodes represent genes and links represent protein-protein interaction of gene products. Nodes were colored according to the pathways they belong. Nodes with more than one color belong to more than one pathway evaluated.

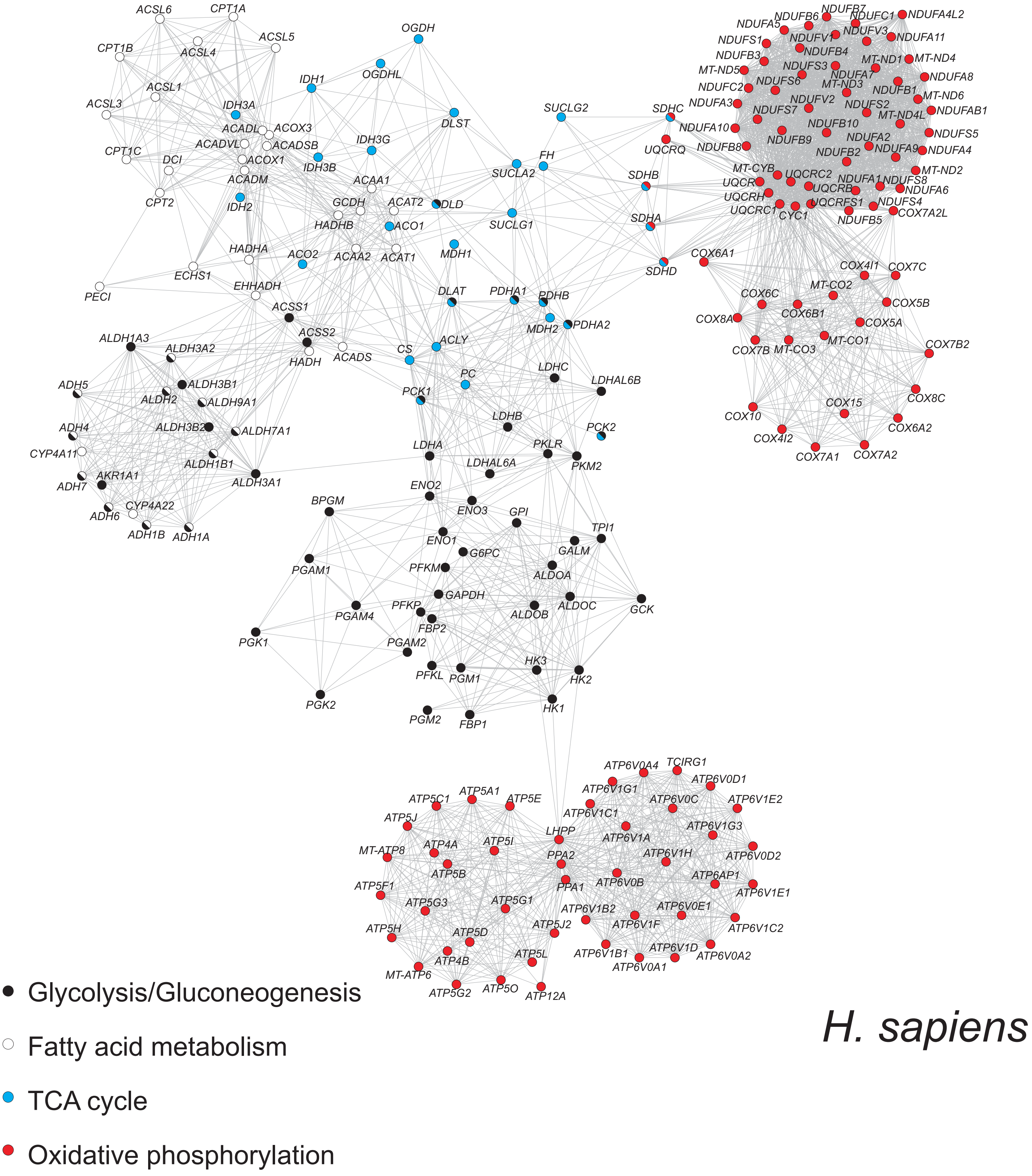

Supplementary Figure S9. *H. sapiens* energetic metabolism gene network. Energetic metabolism protein-protein interaction network of *H. sapiens* is shown. Nodes represent genes and links represent protein-protein interaction of gene products. Nodes were colored according to the pathways they belong. Nodes with more than one color belong to more than one pathway evaluated.

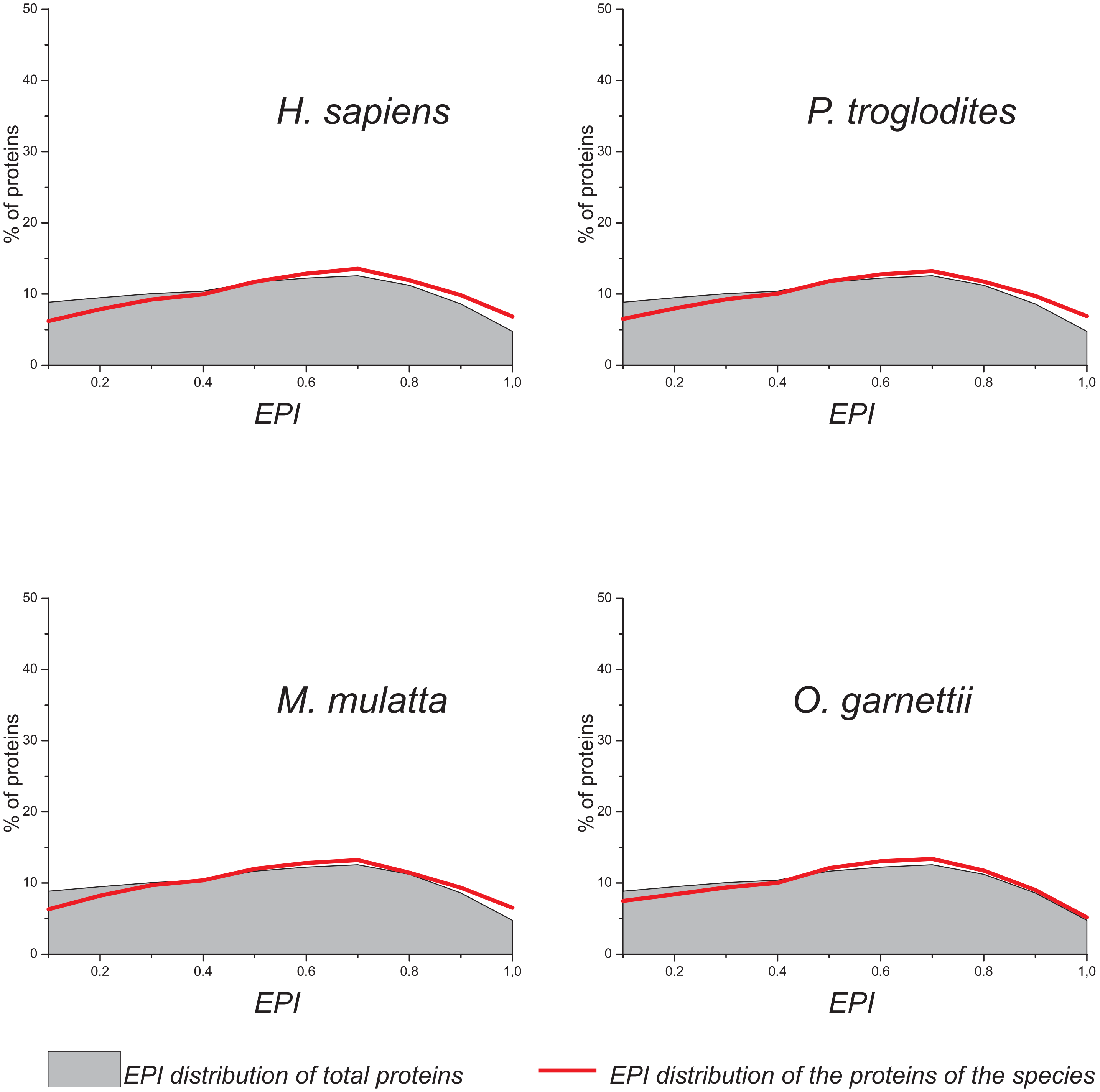

Supplementary Figure S10. *EPI* distribution of different species. Grey landscape represents the *EPI* distribution of all proteins present in KOG dataset and red line represents the *EPI* distribution of all proteins of each species.

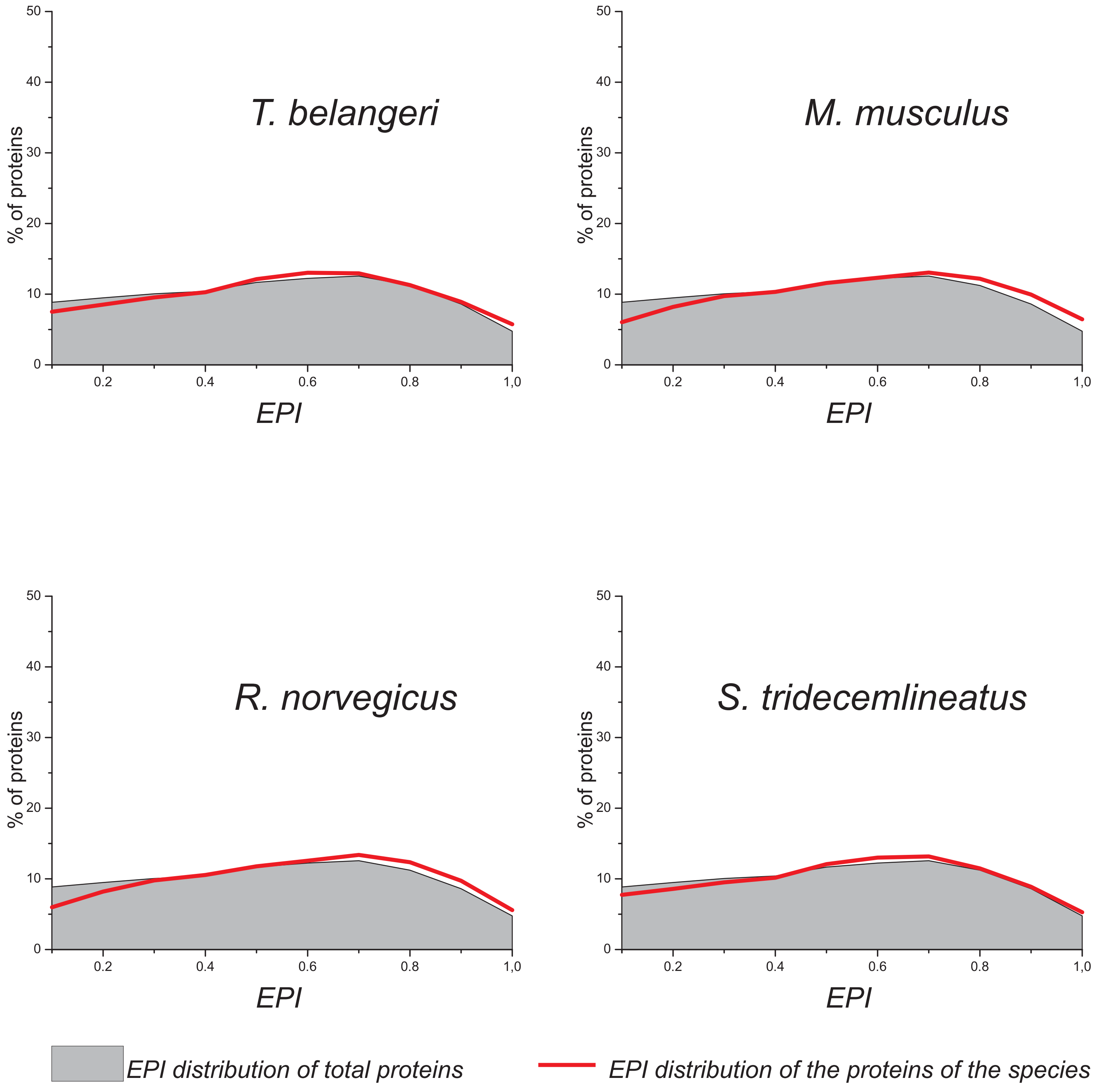

Supplementary Figure S11. *EPI* distribution of different species. Grey landscape represents the *EPI* distribution of all proteins present in KOG dataset and red line represents the *EPI* distribution of all proteins of each species.

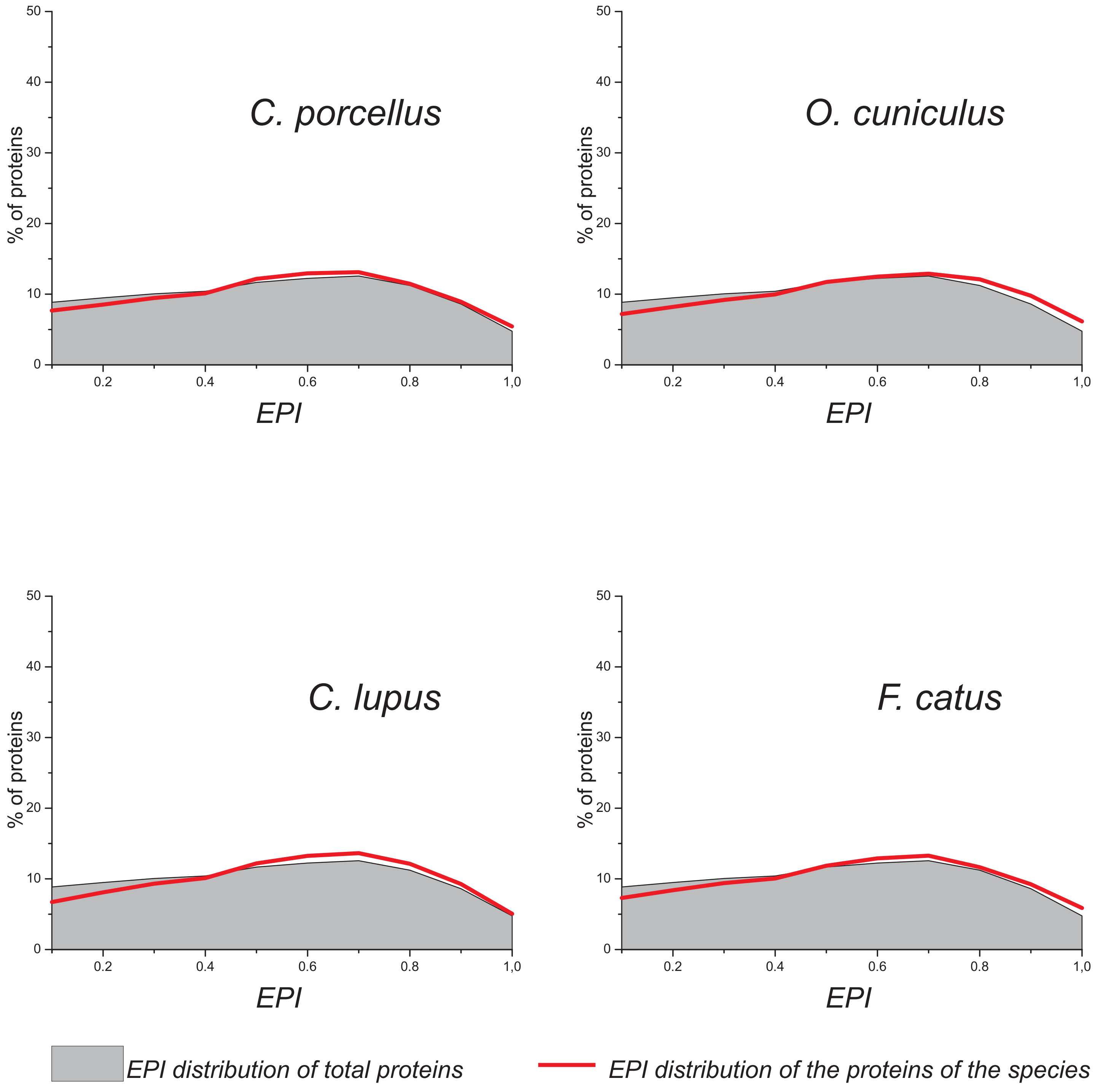

Supplementary Figure S12. *EPI* distribution of different species. Grey landscape represents the *EPI* distribution of all proteins present in KOG dataset and red line represents the *EPI* distribution of all proteins of each species.

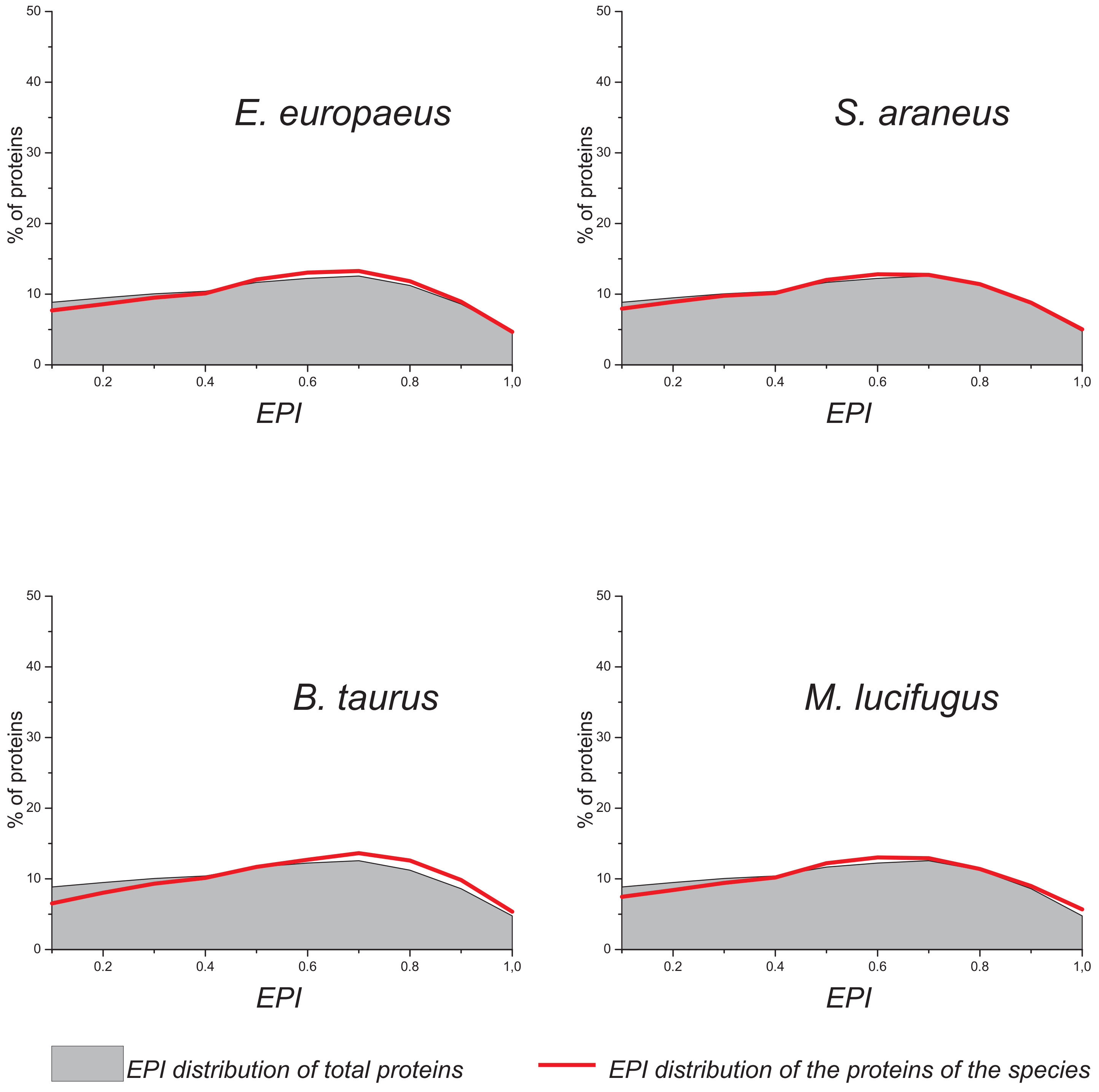

Supplementary Figure S13. *EPI* distribution of different species. Grey landscape represents the *EPI* distribution of all proteins present in KOG dataset and red line represents the *EPI* distribution of all proteins of each species.

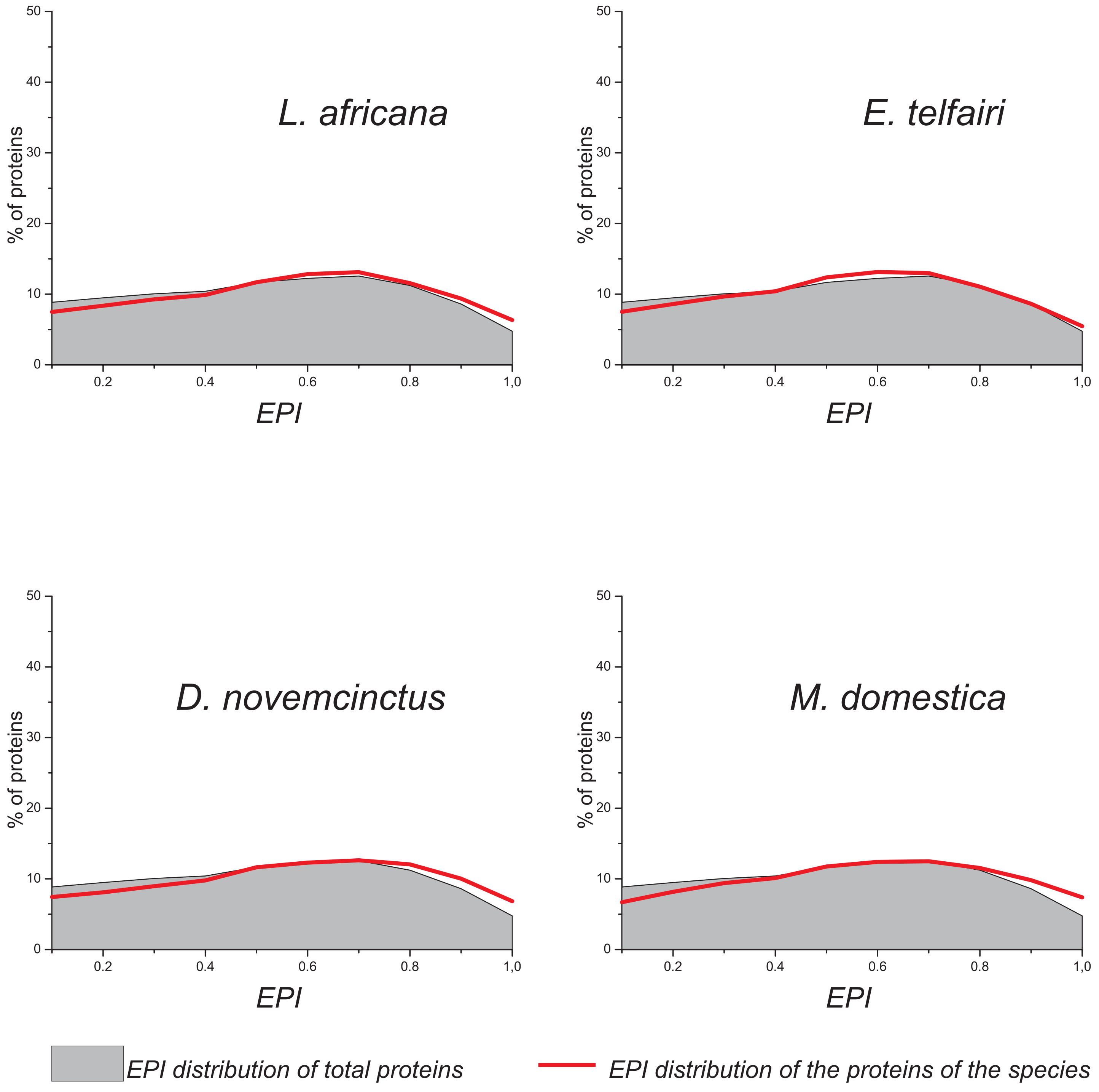

Supplementary Figure S14. *EPI* distribution of different species. Grey landscape represents the *EPI* distribution of all proteins present in KOG dataset and red line represents the *EPI* distribution of all proteins of each species.

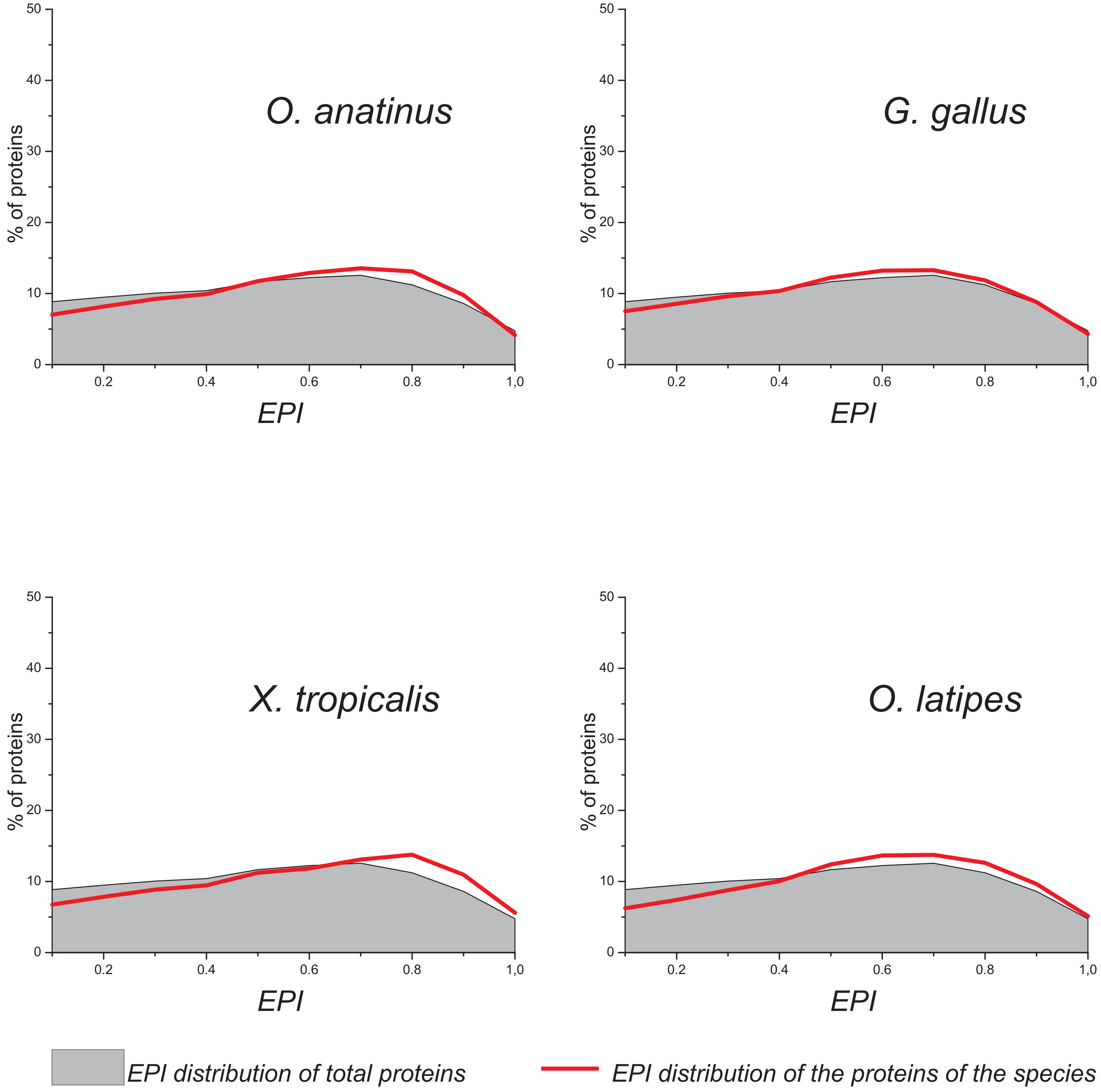

Supplementary Figure S15. *EPI* distribution of different species. Grey landscape represents the *EPI* distribution of all proteins present in KOG dataset and red line represents the *EPI* distribution of all proteins of each species.

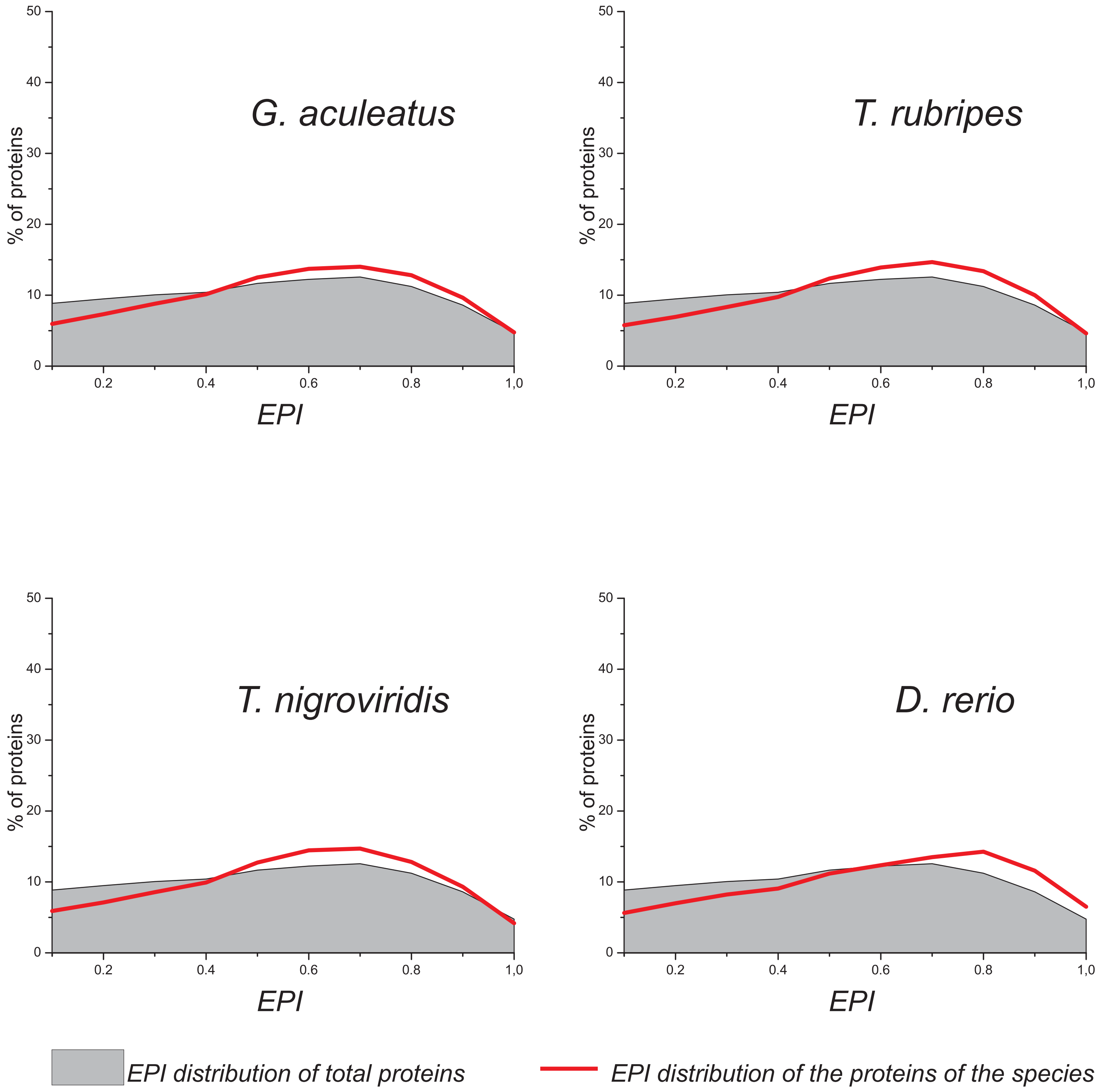

Supplementary Figure S16. *EPI* distribution of different species. Grey landscape represents the *EPI* distribution of all proteins present in KOG dataset and red line represents the *EPI* distribution of all proteins of each species.

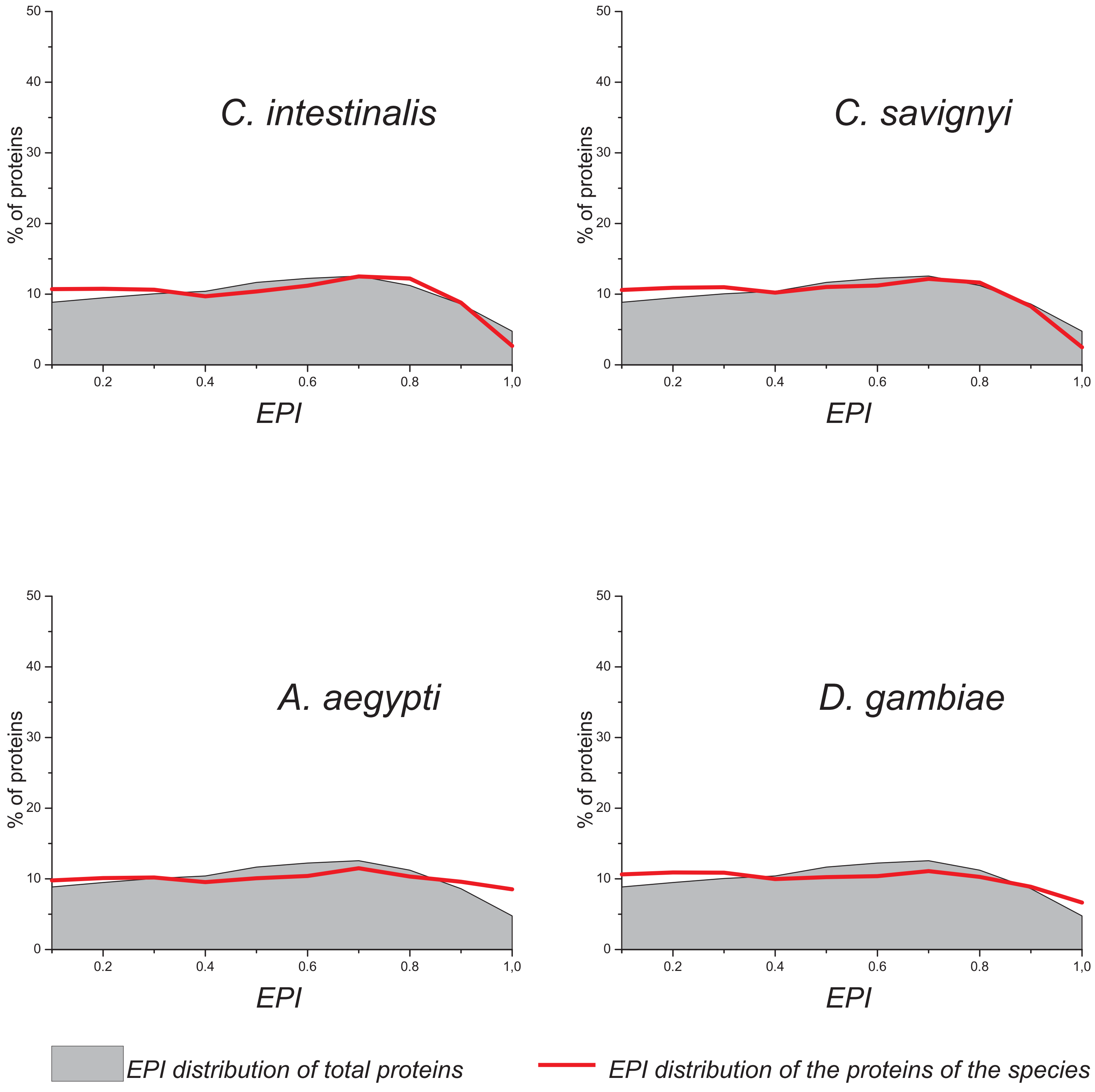

Supplementary Figure S17. *EPI* distribution of different species. Grey landscape represents the *EPI* distribution of all proteins present in KOG dataset and red line represents the *EPI* distribution of all proteins of each species.

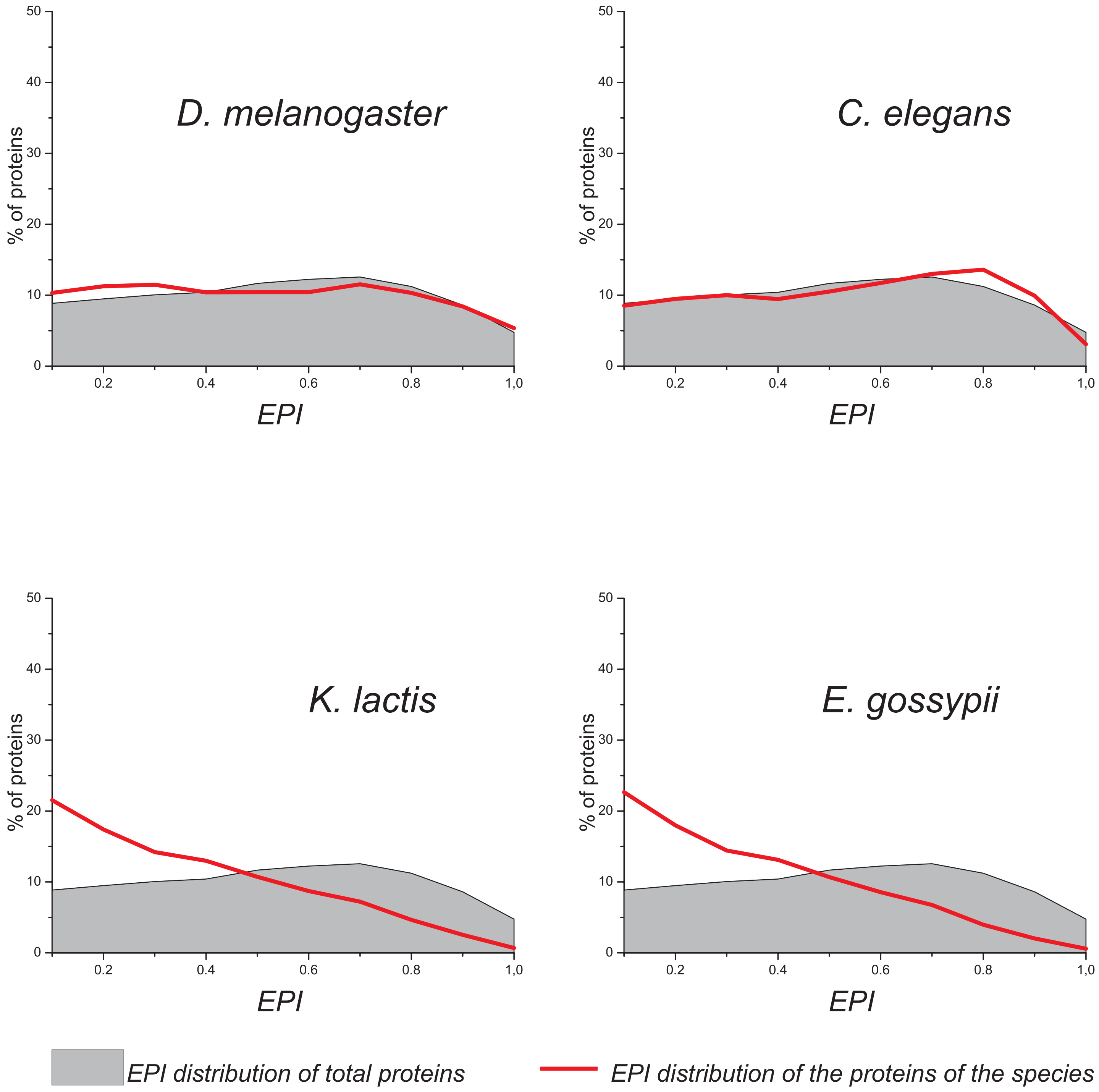

Supplementary Figure S18. *EPI* distribution of different species. Grey landscape represents the *EPI* distribution of all proteins present in KOG dataset and red line represents the *EPI* distribution of all proteins of each species.

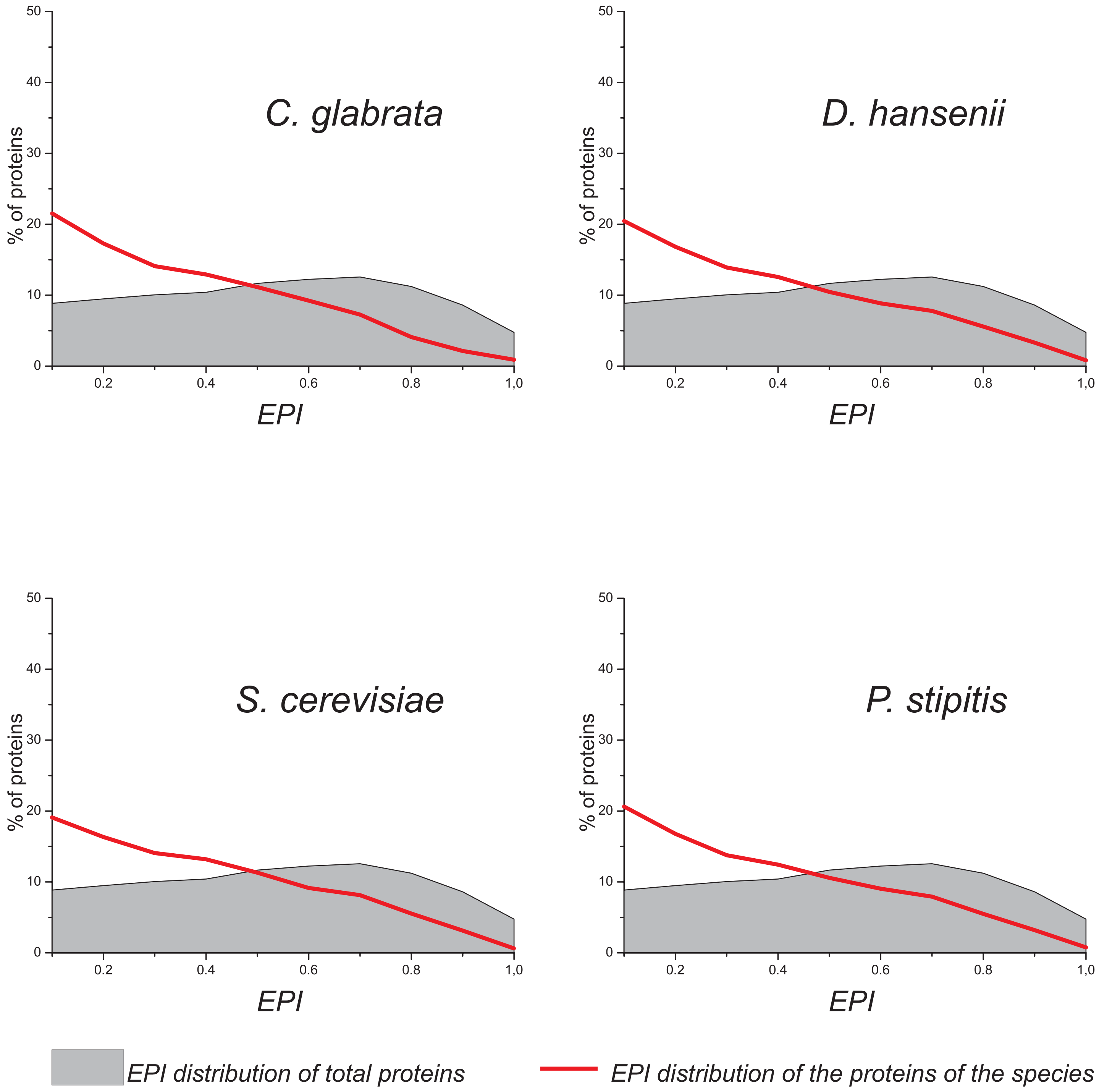

Supplementary Figure S19. *EPI* distribution of different species. Grey landscape represents the *EPI* distribution of all proteins present in KOG dataset and red line represents the *EPI* distribution of all proteins of each species.

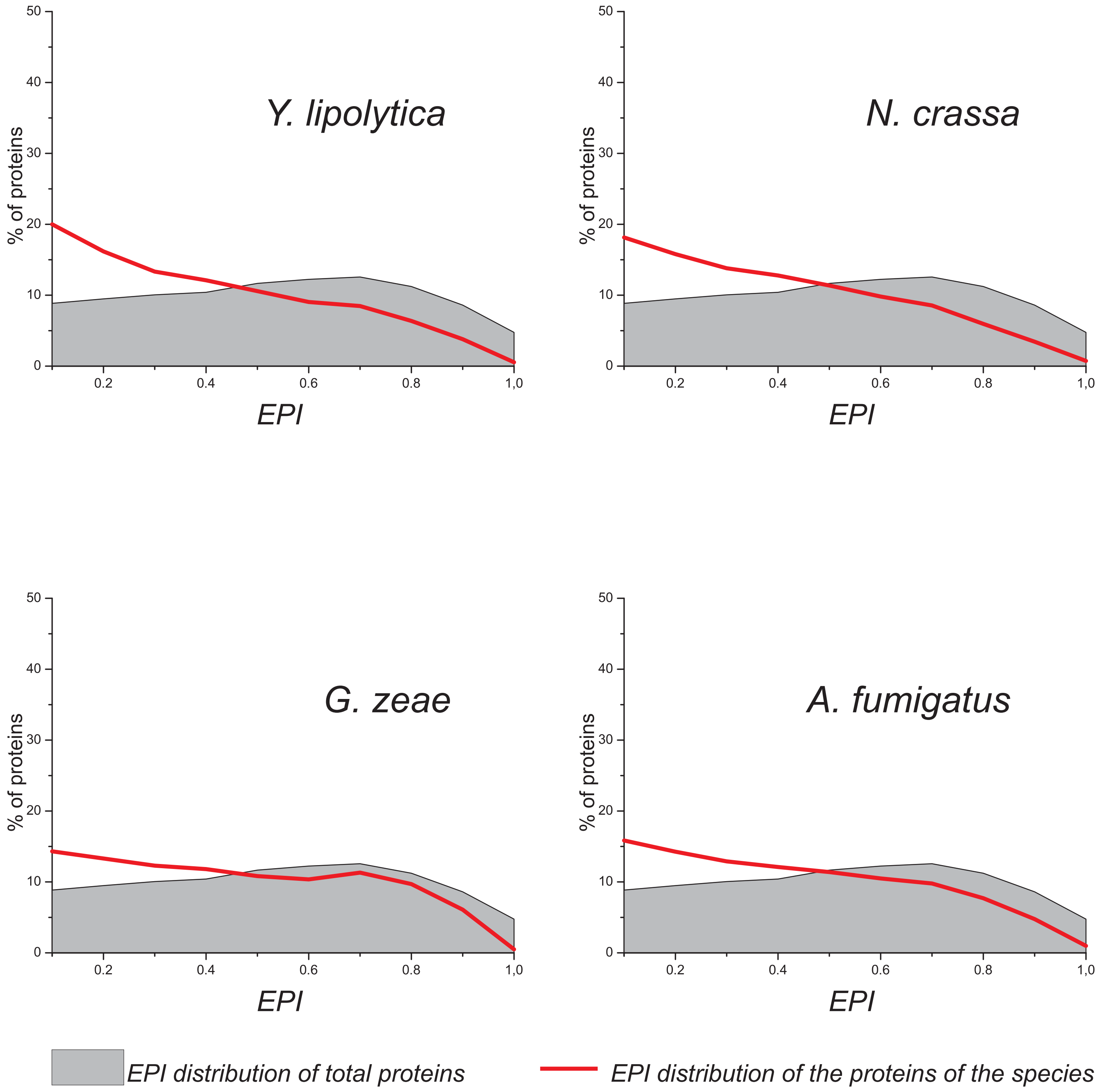

Supplementary Figure S20. *EPI* distribution of different species. Grey landscape represents the *EPI* distribution of all proteins present in KOG dataset and red line represents the *EPI* distribution of all proteins of each species.

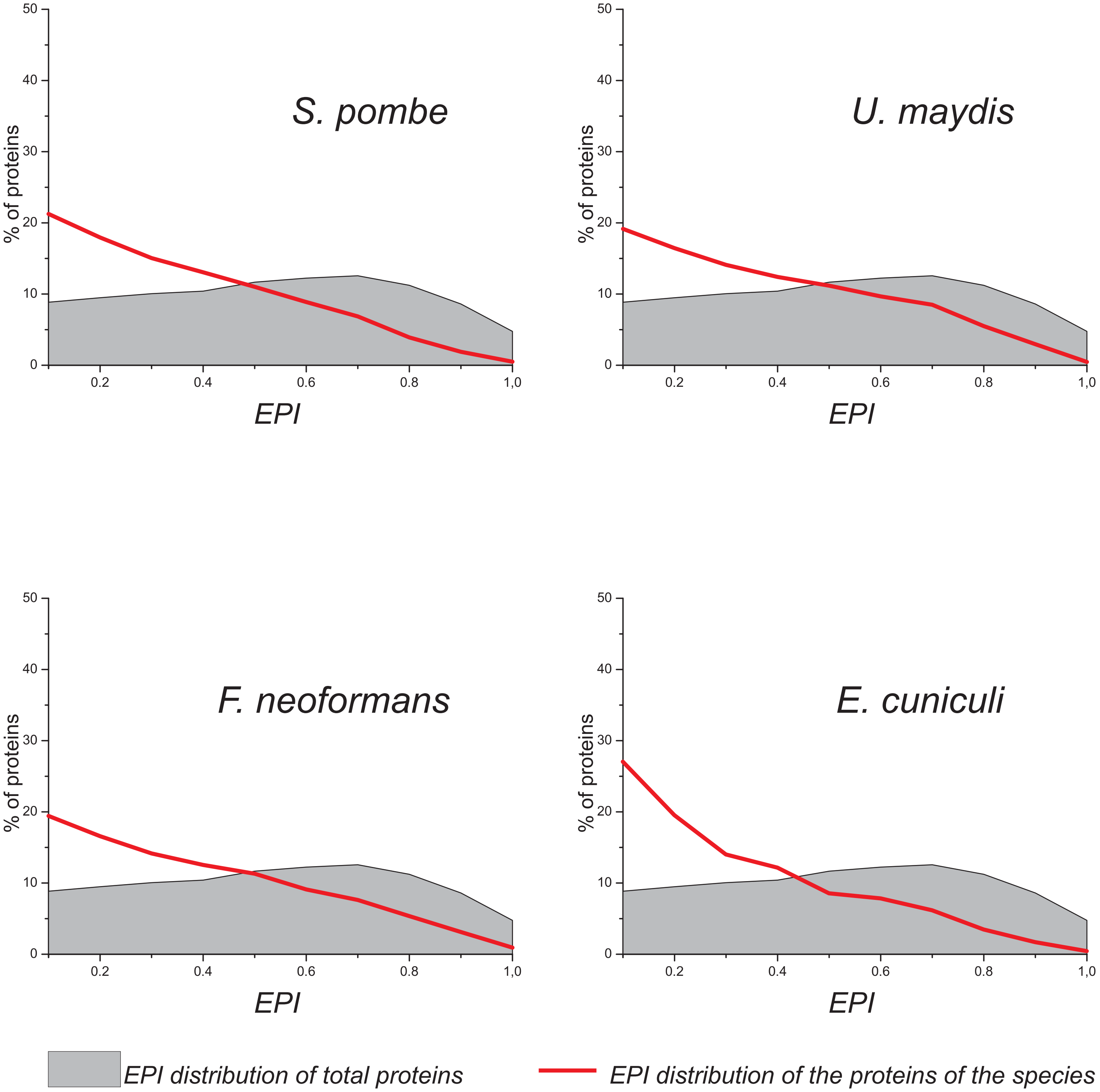

Supplementary Figure S21. *EPI* distribution of different species. Grey landscape represents the *EPI* distribution of all proteins present in KOG dataset and red line represents the *EPI* distribution of all proteins of each species.

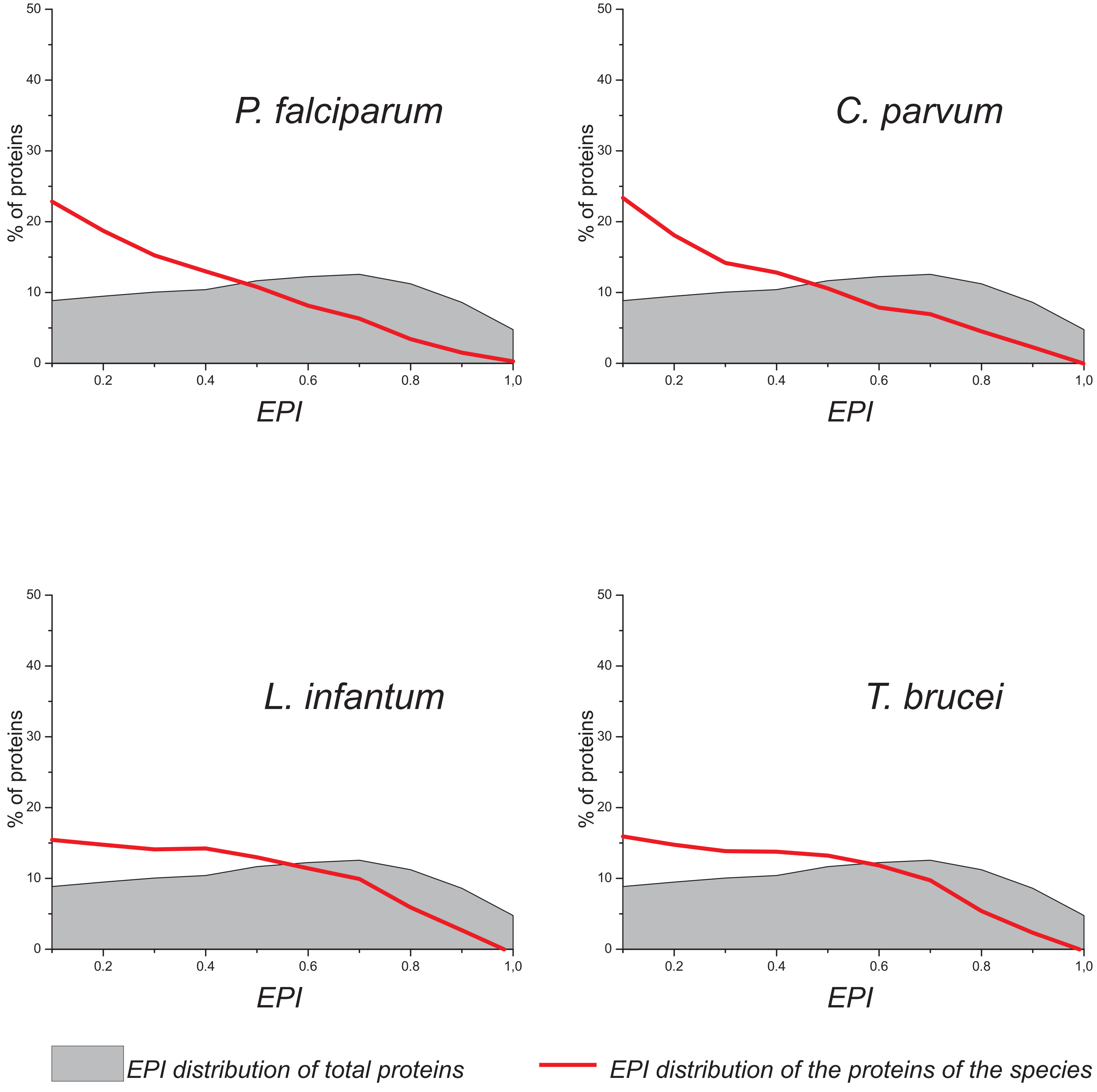

Supplementary Figure S22. *EPI* distribution of different species. Grey landscape represents the *EPI* distribution of all proteins present in KOG dataset and red line represents the *EPI* distribution of all proteins of each species.

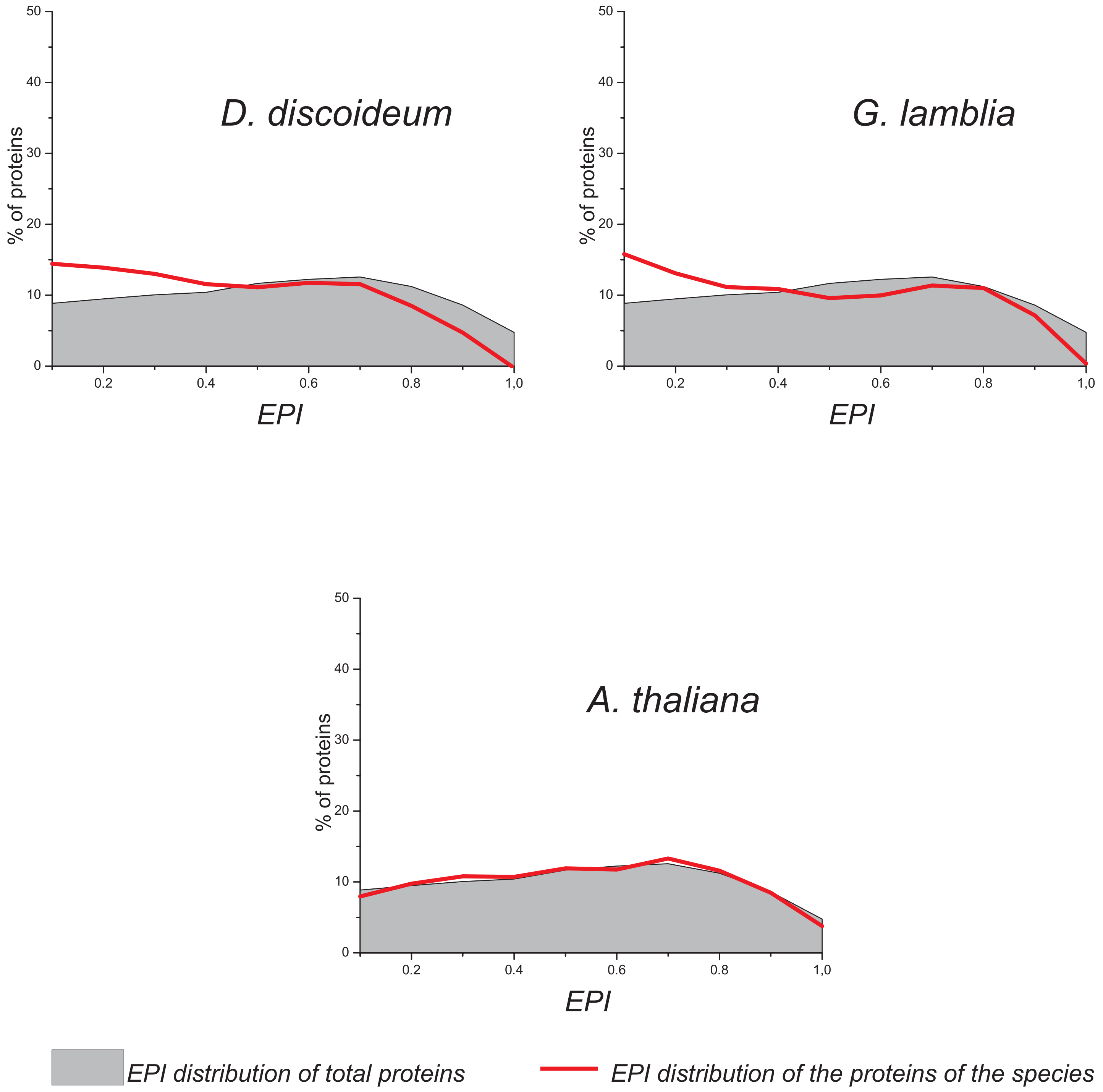

Supplementary Figure S23. *EPI* distribution of different species. Grey landscape represents the *EPI* distribution of all proteins present in KOG dataset and red line represents the *EPI* distribution of all proteins of each species.

2. References

1. Kanehisa M, Goto S: **KEGG: Kyoto Encyclopedia of Genes and Genomes.** *Nucl Acids Res* 2000, **28:** 27-30.

2. Tatusov R, Fedorova N, Jackson J, Jacobs A, Kiryutin B, Koonin E *et al.*: **The COG database: an updated version includes eukaryotes.** *BMC Bioinformatics* 2003, **4:** 41.

3. O'Brien KP, Remm M, Sonnhammer ELL: **Inparanoid: a comprehensive database of eukaryotic orthologs.** *Nucl Acids Res* 2005, **33:** D476-D480.

4. Koonin EV, Makarova KS, Aravind L: **HORIZONTAL GENE TRANSFER IN PROKARYOTES: Quantification and Classification1.** *Annual Review of Microbiology* 2001, **55:** 709-742.

5. Jensen LJ, Kuhn M, Stark M, Chaffron S, Creevey C, Muller J *et al.*: **STRING 8--a global view on proteins and their functional interactions in 630 organisms.** *Nucl Acids Res* 2009, **37:** D412-D416.

6. Dotsch A, Klawonn F, Jarek M, Scharfe M, Blocker H, Haussler S: **Evolutionary conservation of essential and highly expressed genes in Pseudomonas aeruginosa.** *BMC Genomics* 2010, **11:** 234.

7. He X, Zhang J: **Higher Duplicability of Less Important Genes in Yeast Genomes.** *Mol Biol Evol* 2006, **23:** 144-151.

8. Liao BY, Scott NM, Zhang J: **Impacts of Gene Essentiality, Expression Pattern, and Gene Compactness on the Evolutionary Rate of Mammalian Proteins.** *Mol Biol Evol* 2006, **23:** 2072-2080.

9. Liao BY, Zhang J: **Mouse duplicate genes are as essential as singletons.** *Trends in Genetics* 2007, **23:** 378-381.

10. Carroll SB: **Chance and necessity: the evolution of morphological complexity and diversity.** *Nature* 2001, **409:** 1102-1109.

11. Castro MA, Dalmolin RJ, Moreira JC, Mombach JC, de Almeida RM: **Evolutionary origins of human apoptosis and genome-stability gene networks.** *Nucleic Acids Res* 2008, **36:** 6269-6283.

12. Chen S, Zhang YE, Long M: **New Genes in Drosophila Quickly Become Essential.** *Science* 2010, **330:** 1682-1685.

13. Aravind L, Walker DR, Koonin EV: **Conserved domains in DNA repair proteins and evolution of repair systems.** *Nucl Acids Res* 1999, **27:** 1223-1242.

14. Igaki T, Kanda H, Yamamoto-Goto Y, Kanuka H, Kuranaga E, Aigaki T *et al*.: **Eiger, a TNF superfamily ligand that triggers the Drosophila JNK pathway.** *EMBO Journal* 2002, **21:** 3009-3018.
